# Physiological and Molecular Characterization of an Oxidative Stress-Resistant *Saccharomyces cerevisiae* Strain Obtained by Evolutionary Engineering

**DOI:** 10.3389/fmicb.2022.822864

**Published:** 2022-02-24

**Authors:** Nazlı Kocaefe-Özşen, Bahtiyar Yilmaz, Ceren Alkım, Mevlüt Arslan, Alican Topaloğlu, Halil l̇brahim Kısakesen, Erdinç Gülsev, Z. Petek Çakar

**Affiliations:** ^1^Department of Molecular Biology and Genetics, Istanbul Technical University, Istanbul, Turkey; ^2^Dr. Orhan Öcalgiray Molecular Biology, Biotechnology and Genetics Research Center (ITU-MOBGAM), Istanbul Technical University, Istanbul, Turkey

**Keywords:** oxidative stress, reactive oxygen species, evolutionary engineering, stress resistance, heat preconditioning, *Saccharomyces cerevisiae*, adaptive laboratory evolution, genomic variants

## Abstract

Oxidative stress is a major stress type observed in yeast bioprocesses, resulting in a decrease in yeast growth, viability, and productivity. Thus, robust yeast strains with increased resistance to oxidative stress are in highly demand by the industry. In addition, oxidative stress is also associated with aging and age-related complex conditions such as cancer and neurodegenerative diseases. *Saccharomyces cerevisiae*, as a model eukaryote, has been used to study these complex eukaryotic processes. However, the molecular mechanisms underlying oxidative stress responses and resistance are unclear. In this study, we have employed evolutionary engineering (also known as adaptive laboratory evolution – ALE) strategies to obtain an oxidative stress-resistant and genetically stable *S. cerevisiae* strain. Comparative physiological, transcriptomic, and genomic analyses of the evolved strain were then performed with respect to the reference strain. The results show that the oxidative stress-resistant evolved strain was also cross-resistant against other types of stressors, including heat, freeze-thaw, ethanol, cobalt, iron, and salt. It was also found to have higher levels of trehalose and glycogen production. Further, comparative transcriptomic analysis showed an upregulation of many genes associated with the stress response, transport, carbohydrate, lipid and cofactor metabolic processes, protein phosphorylation, cell wall organization, and biogenesis. Genes that were downregulated included those related to ribosome and RNA processing, nuclear transport, tRNA, and cell cycle. Whole genome re-sequencing analysis of the evolved strain identified mutations in genes related to the stress response, cell wall organization, carbohydrate metabolism/transport, which are in line with the physiological and transcriptomic results, and may give insight toward the complex molecular mechanisms of oxidative stress resistance.

## Introduction

In all aerobic organisms, the generation of reactive oxygen species (ROS), as side products of cellular metabolism, including hydrogen peroxide (H_2_O_2_), the hydroxyl radical (.OH), or superoxide anions (O_2_^–^) by reduction of molecular oxygen is an inevitable aspect of life (Jamieson, 1992; Izawa et al., 1995; Bayliak et al., 2017). All living organisms including microbes, plants and other multicellular organisms use signal transduction mechanisms to sense and respond to different forms of environmental stress (Hong et al., 2013). As plants are sedentary organisms and possess photosynthetic systems, they cannot move to find optimal conditions and consequently, they produce a lot of ROS. They balance the overoxidation and overreduction with short term and long-term mechanisms, including enzymatic systems (Lushchak, 2011). In mammalian systems, about 90% of the ATP is generated *via* oxidative phosphorylation and is the primary source of ROS in cells (Candas and Li, 2014). An increasing body of evidence indicates that oxidative stress participates in the pathogenesis of many diseases including cardiovascular disease (Förstermann, 2008), cancer (Reuter et al., 2010), neurodegenerative disorders (Li et al., 2013), and inflammatory bowel diseases (Lih-Brody et al., 1996; Sokol et al., 2017). When antioxidant defenses are overwhelmed and unable to counteract ROS, the resulting oxidative stress (Finkel and Holbrook, 2000; Finkel, 2005) can damage nucleic acids, oxidize amino acids, as well as co-factors of proteins, and disturb cellular homeostasis (Imlay, 2015).

Reactive oxygen species are also specifically generated in host phagocytes as microbicidal molecules and in non-phagocytic cells they have important roles in fertilization, thyroid hormone synthesis and cell signaling (Görlach et al., 2015). These products of oxygen metabolism may have damaging effects on an organism, in essence, due to oxidation of essential cellular components. Steady-state formation of pro-oxidant free radicals is normally balanced by a similar rate of breakdown by antioxidants (Finkel and Holbrook, 2000; Finkel, 2005). Although imperfectly coupled aerobic respiration is the main source of ROS, they can also be generated by peroxisomal β-oxidation of fatty acids, microsomal cytochrome P450 metabolism of xenobiotic compounds, stimulation of phagocytic NADP-oxidase by pathogens or lipopolysaccharides, and tissue specific enzymes. In an inflammatory condition, the host cannot maintain anaerobic conditions in the gut, consequently leading to reductions in microbiota-derived fermentation products such as short-chain fatty acids (SCFAs) produced by obligate anaerobic bacteria in the colon: these are important to promote the maturation and expansion of regulatory T (T_reg_) cells (Espey, 2013; Faber and Bäumler, 2014; Byndloss et al., 2017). The amount of oxygen diffusion into the gut lumen is limited by epithelial metabolism and thus, obligate anaerobic bacteria are able to continue short chain fatty acid production in the intestinal lumen. In contrast, inflammation leads to the loss of butyrate, which disrupts metabolic signaling in intestinal epithelial cells and this promotes nitrate in the lumen *via* nitric oxidase induction and also disables β-oxidation in epithelial cells that would otherwise control the oxygen levels in the colon (Byndloss et al., 2017; Rivera-Chávez et al., 2017). ROS, that plays a critical role in host-microbe interactions, may lead to cellular oxidative damage that makes host tissues more susceptible to oxidant-induced damage.

Antioxidant mechanisms provide an evolutionary advantage to cells to control ROS production and its deleterious effects. There have been numerous studies characterizing the response mechanism of living organisms to oxidative stress, which has been mostly studied in the prokaryotes *Escherichia coli* and *Salmonella typhimurium*. Many proteins and their corresponding genes in bacterial defense systems against oxidative stress have been identified (Storz et al., 1990). As a eukaryotic model organism, *Saccharomyces cerevisiae* has been useful to study the response to endogenous and exogenous oxidative stress. It possesses several enzymatic and non-enzymatic ROS detoxifying enzymes, such as cytochrome *c* peroxidase, superoxide dismutase (SOD), glutathione (GSH) peroxidase, catalase (CAT), glutaredoxin and peroxiredoxin to protect cellular compartments and maintain a cellular redox state (Jamieson, 1998; Charizanis et al., 1999; Park et al., 2000; Grant, 2001).

The heat stress response, a protective transcriptional program activation against heat stress, includes the reprogramming of an important part of the transcriptome. Environmental Stress Response (ESR) genes, involved in metabolism, oxidant defense, and growth control, are induced by the heat shock (Davidson et al., 1996). There is an important overlap between the gene expression programs against different stress factors. This phenomenon is named “cross-protection.” Treatment with one stress enhances tolerance to a subsequent stressor of a different nature; e.g., heat shock induces tolerance to oxidants or osmotic shock. Specifically, genes that are regulated by the transcription factors Msn2/4, most of which exhibit a common gene expression profile in response to various stressors, suggest a general, rather than a stress-specific response. Nowadays, heat stress and oxidative stress responses are better understood. The focus now is on the products generated by the stress response and their roles in stress tolerance and adaptation. Although the expression of the genes related to stress response have been studied, the precise signaling mechanism that triggers this reaction remains unclear (Morano et al., 2012).

The definition of heat stress is a rise in temperature beyond a threshold level for a period sufficient to cause irreversible damage within a few minutes to cellular compartments on the host as a consequence of extensive protein denaturation and aggregation, and loss of membrane integrity (Wahid, 2007; Allakhverdiev et al., 2008). Oxidative stress is directly linked to heat stress and when it occurs simultaneously with heat exposure, it can manifest in all parts of the body. Upon heat stress, ROS and NADPH oxidase levels in different cellular compartments such as chloroplasts, mitochondria and peroxisomes increase as a by-product in various aerobic metabolic pathways, and the antioxidant enzyme activities are down-regulated (Morimoto, 1998). In a chronic heat stress condition, downsizing of mitochondrial metabolic oxidative capacity, up-regulation of uncoupling proteins, a clear reduction in antioxidant enzyme activities, and depletion of antioxidant reserves occur, as the main reasons for the reduced tolerance to oxidative stress and tissue damage.

The very first step of induction of heat stress appears to be an increase in cellular energy expenditure and later, enhanced mitochondrial transportation and β-oxidation of fatty acids. To cope with the enhanced energy expenditure within cells and mitochondrial biogenesis, the production of reducing equivalents and the enzymatic activity of subunits of respiratory chain complexes are increased. Increased mitochondrial energy generation is intrinsically associated with an increase in ROS (Mujahid et al., 2005; Yang et al., 2010). Defense mechanisms against oxidative and heat stress conditions share a common player. Induced protection for heat stress tolerance has been suggested to be correlated with the appearance of heat shock proteins which is also induced by H_2_O_2_, UV radiation, sodium arsenite and cadmium, all common oxidative stress inducers in mammalian systems. In mammals, HO-1 (heme oxygenase – 1) can also be induced, which are protective against these stress conditions (Steels et al., 1992). In contrast, the major players in yeast cells against heat and oxidative stress are the heat shock transcription factor 1 (HSF) protein family, the primary modulator of the heat shock response, and Msn2/4 – all stress-responsive transcriptional activators. Besides, mild heat shock can activate the cell wall integrity pathway which can give an advantage for further activation of Hsf1 or Msn2/4 (Levin, 2005; Morano et al., 2012).

Evolutionary engineering, also known as adaptive laboratory evolution (ALE), is an experimental strategy mimicking nature’s own engineering principle *via* variation and selection. Evolutionary selection principles have been used for the improvement of biotechnologically important characteristics, including novel catabolic activities, improved enzyme properties, plasmid functions, and stress resistance (Sauer, 2001; Çakar et al., 2005, 2012). *S. cerevisiae* strains that are resistant against diverse stress types have been successfully obtained by evolutionary engineering, including nickel-resistant (Küçükgöze et al., 2013), cobalt-resistant (Çakar et al., 2009; Alkim et al., 2013), iron-resistant (Balaban et al., 2020), coniferyl aldehyde-resistant (Hacısalihoğlu et al., 2019), caffeine-resistant (Surmeli et al., 2019), ethanol-tolerant (Turanlı-Yıldız et al., 2017), chronologically long-lived (Arslan et al., 2018a,b), and silver-resistant (Terzioğlu et al., 2020) *S. cerevisiae*.

The major advantage of evolutionary engineering is that it does not require any detailed prior information about the desired phenotypes. The only requirement is a selection scheme that will favor the desired phenotype (Sauer, 2001). Thus, it is highly advantageous to obtain genetically complex phenotypes, such as oxidative stress-resistant yeasts. Any selective condition that may show an oxidative stress effect such as H_2_O_2_ can be used as the selective pressure. Another advantage of evolutionary engineering is the ability to analyze evolved strains at genomic and transcriptomic levels, owing to the rapid development in omics technologies. This allows identification of the key changes in the evolved strains that are associated with the desired phenotype (Çakar et al., 2012). Despite the various studies on oxidative stress responses and resistance, the complex molecular mechanisms are unclear. Further, to the best of our knowledge, there are no studies on oxidative stress-resistant *S. cerevisiae* strains obtained by evolutionary engineering and their characterization. Moreover, to our knowledge, there are also no reports comparing the efficiencies of different evolutionary selection strategies in obtaining oxidative stress-resistant *S. cerevisiae* strains, particularly to elucidate the role of heat and oxidative pretreatment.

In this study, we first generated a genetically stable, oxidative stress-resistant *S. cerevisiae* strain using evolutionary engineering strategies. These included gradually increased and continuously applied selective oxidative stress levels over populations of selection, with and without heat and oxidative stress pretreatment. Prior to the evolutionary engineering selection, yeast cells were treated with the chemical mutagen ethyl methane sulfonate (EMS), to increase the genetic diversity of the initial population of selection by random mutagenesis. The phenotypic and physiological characteristics and the transcriptomic profile of the evolved strain showed that the acetate consumption and maltose accumulation increased, with elevated gene expression levels related to stress response; carbohydrate, lipid, and protein metabolic processes including anabolic and catabolic reactions; generation of precursor metabolites and energy; transportation and autophagy. In addition, we found down-regulated genes that were associated with nuclear transport, organelle assembly, tRNA, cell cycle function and mitosis. The genomic profile of the evolved strain showed that mutations mainly accumulated in genes directly associated with the stress response, cell wall organization, and carbohydrate metabolism/transport, which are in accordance with the physiological and transcriptomic results. Overall, our study shows how yeast can evolve by altering its genomic profile to cope with an experimental oxidative stress condition and how these genetic alterations can be beneficial in different stress environments. It also indicates the key molecular factors that are associated with oxidative stress resistance in yeast.

## Materials and Methods

### Strain, Media, and Growth Conditions

The *S. cerevisiae* CEN.PK 113-7D strain (MATa, MAL2-8c, SUC2), kindly provided by Prof. Dr. Jean Marie François and Dr. Laurent Benbadis (University of Toulouse, France), was used as the reference strain (*905*). Unless otherwise stated, yeast cultivations were performed in yeast minimal medium (YMM), containing 0.67% “w/v” yeast nitrogen base without amino acids (Difco, New Jersey, United States) and 2.0% (w/v) glucose (VWR BDH PROLABO, Leicestershire, United Kingdom) as the sole carbon source, under aerobic conditions at 30°C. Cell growth was monitored spectrophotometrically by optical density measurements at 600 nm (OD_600_).

Ethyl methane sulfonate (Sigma, St. Louis, MO, United States) mutagenesis of the reference strain was carried out as described previously (Lawrence, 1991; Balaban et al., 2020), under conditions that resulted in a 10% survival rate after treatment to obtain a randomly mutated initial *S. cerevisiae* population (*906*) with increased genetic diversity. That population was used directly as the starting population for all selection experiments.

### Selection Strategy for Obtaining Evolutionary Engineered–Continuous Oxidative Stress Resistant Yeast Cells

**FIGURE 1:**
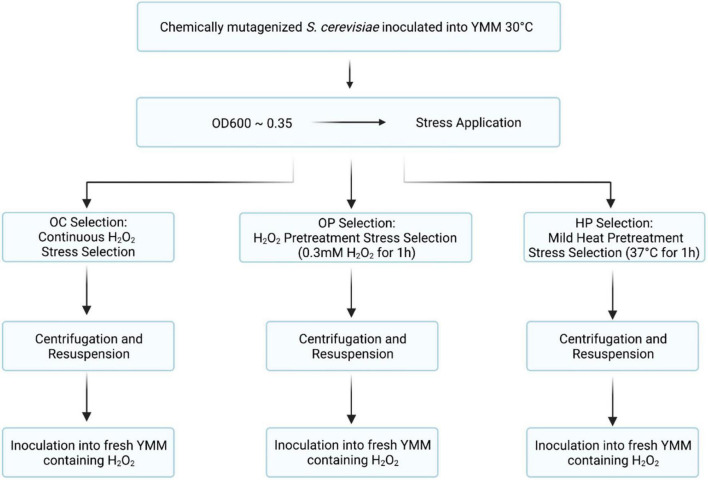
Flow charts of evolutionary selection and preconditioning strategies. Generation and selection of oxidative stress-resistant *S. cerevisiae* populations using different evolutionary engineering strategies are shown (OC, oxidative continuous; OP, oxidative pulse; HP, heat and pulse).

Three different evolutionary engineering strategies were employed to select for oxidative stress-resistant evolved strains (Figure 1).

i Oxidative continuous (OC) stress selection: the EMS-mutagenized *S. cerevisiae* population called *906* was initially cultivated in YMM containing 0.5-mM H_2_O_2_ and the successive population surviving that oxidative stress was then exposed to increasing H_2_O_2_ levels, up to 10 mM H_2_O_2_. Each successive population was named as OC X*^th^* Population (O and C stand for Oxidative and Continuous, respectively and X shows the population number).
ii Oxidative pulse (OP) stress pretreatment selection: the EMS-mutagenized *S. cerevisiae 906* population was initially cultivated in YMM containing 0.3-mM H_2_O_2_ and incubated for 1 h as a pulse oxidative stress. Our initial pulse oxidative stress tests with the *906* culture showed that there was about 30% decrease in survival upon 0.3-mM H_2_O_2_ stress. Thus, 0.3-mM H_2_O_2_ was used as a mild, but effective stress level for oxidative stress pre-conditioning. Following pre-conditioning, the culture was subjected to continuous oxidative stress as in (i). Each successive population was named as OP X*^th^* Population (O and P stand for Oxidative and Pulse, respectively and X shows the population number). The pre-conditioning step was repeated at each successive population of selection.
iii Heat pulse (HP) stress pretreatment selection: the EMS-mutagenized *S. cerevisiae 906* population was initially incubated in YMM at 37°C for 1 h as a pulse heat stress. It was then subjected to continuous oxidative stress as in (i). Each successive population was named as HP X*^th^* Population (H and P stand for Heat and Pulse, respectively and X shows the population number). The pre-conditioning step was repeated at each successive population of selection.

During these selection experiments, for each population a parallel control group was incubated in YMM at 30°C. At the end of each selection experiment, 10 randomly chosen individual strains were picked from solid YMM plates of the final populations and the cultures were stored at –80°C in 30% “v/v” glycerol.

### Estimation of Stress Resistance Levels of the Evolved Strains

For the quantitative estimation of stress resistance, viable cell counts were made both by the most probable number (MPN) method and colony counting on YMM agar plates, as described previously (Russek and Colwell, 1983; Çakar et al., 2005). For the MPN method, dilutions were made in the range of 10^–1^ to 10^–8^ for five parallel samples in 96-well plates and the MPN of cells surviving the stress condition was estimated, as described previously (Çakar et al., 2005). In addition, cultures grown in the absence of any stress condition were used as controls. MPN scores were determined after 48 and 72 h of incubation. Each score refers to the “number of organisms per unit volume” of the original sample and was calculated from the tables indicated (Lindquist, 2016). The survival rates of the cultures were determined by calculating the ratio between the number of cells/mL under stress condition and those under control conditions.

To estimate the oxidative stress resistance levels, cells were cultivated until their mid-logarithmic phase (OD_600_ of 1–1.5) and were subjected to either continuous oxidative stress (YMM containing 1-mM and 2-mM H_2_O_2_, 72 h, 30^°^C) or pulse oxidative stress (YMM containing 0.1 M and 0.3 M H_2_O_2_ for 90 min followed by incubation in YMM, 72 h, 30^°^C). The survival rates were then determined by calculating the ratio between the number of viable cells in stress conditions and that under control conditions.

The cross-resistances to other stress conditions such as CoCl_2_, salt, ethanol, heat, and freeze-thaw stress were also determined using the same method for the estimation of oxidative stress resistance. Briefly, the following stress conditions were applied during the cross-resistance tests:

• Heat stress by incubating strains in YMM at 60°C for 10 min,
• Freeze-thaw stress by placing strains in liquid nitrogen and at –20°C for 25 min and thawing at room temperature,
• Ethanol stress by incubating strains in YMM containing 5 and 7% “v/v” ethanol,
• Salt stress by incubating strains in YMM containing 5 and 7% “w/v” NaCl,
• Metal stress by incubating strains in YMM containing 1 mM CoCl_2_.

Additionally, cross-resistances to some other metal and non-metal stress conditions were also determined using the semi-quantitative spot assay by culturing serially diluted samples (up to 10^–5^ dilution) on YMM-agar medium containing a variety of stress factors at a final concentration of CoCl_2_ (1.5-mM), NiCl_2_⋅6H_2_O (0.1-mM), CuSO_4_⋅5H_2_O (0.1-mM), ZnCl_2_⋅6H_2_O (7.5-mM), NaCl (0.5-M), (NH_4_)2Fe(SO_4_)_2_⋅6H_2_O (25-mM), MnSO_4_ (1-mM), and ethanol (8% “v/v”). Resistance profiles were documented upon 72 h incubation at 30°C.

### Physiological Analysis of Evolved Strains Obtained From Different Selection Strategies

Some of the evolved strains that are resistant to continuously applied oxidative stress condition and cross-resistant to other stress conditions were used for further growth physiological analyses, including cell dry weight (CDW) measurements, catalase activity tests, metabolite analysis by high-performance liquid chromatography (HPLC), and trehalose and glycogen analyses.

#### Growth Physiological Experiments and Sample Collection

Overnight cultures of the reference strain and the evolved strain were inoculated in YMM (YMM containing 0-, 0.5-, and 1-mM H_2_O_2_ in 2-L flasks. They were then incubated at 30°C and 200 rpm for 48 h. Samples were withdrawn every 1.5 h for monitoring growth, CDW and metabolites (residual glucose, ethanol, glycerol, acetate, and maltose) and trehalose/glycogen contents.

#### Cell Dry Weight Analysis

A 10 ml cultures of the reference strain and the evolved strain harvested after 48 h of growth in YMM containing 0, 0.5, and 1 mM H_2_O_2_ were used for CDW analysis. After the cell pellets were obtained by centrifugation (5,000 *g*, 5 min), they were dried for 2 h at 90°C, cooled in a desiccator for 30 min and weighed, as described previously (Küçükgöze et al., 2013).

#### Metabolite Analysis by High-Performance Liquid Chromatography

Residual glucose and extracellular metabolites including ethanol, acetate, glycerol, and maltose were analyzed using HPLC, as described previously (Küçükgöze et al., 2013). Briefly, precultures were grown overnight in 100 mL YMM at 30°C and 150 rpm. Cultivations were performed as one control flask containing YMM and two test flasks with YMM containing 0.5 mM and 1 mM H_2_O_2_. The cultures were incubated at 30°C and 150 rpm for 48 h. HPLC samples were prepared by centrifugation of 1.5 mL culture samples at 10,000 g for 5 min, and subsequent filtration of supernatants using 0.2 μm filters. The HPLC system consisted of a system controller (SCL-10A, Shimadzu, Kyoto, Japan), liquid chromatography unit (LC-10AD, Shimadzu, Kyoto, Japan), degasser (DGU-14A, Shimadzu, Kyoto, Japan), refractive index detector (RID-10A, Shimadzu, Kyoto, Japan), auto injector (SIL-10AD, Shimadzu, Kyoto, Japan), and column oven (CTO-10AC, Shimadzu, Kyoto, Japan). An Aminex© HPX-87H column was used at 60°C with a flow rate of 0.6 mL min^–1^, with 20 μL sample injections. 5 mM H_2_SO_4_ solution was used as the mobile phase/eluent (Küçükgöze et al., 2013).

#### Trehalose and Glycogen Determination by Enzymatic Reaction

Reserve carbohydrate (trehalose and glycogen) contents were determined as described previously (Parrou and François, 1997). 25 OD_600_ units of cells were collected, treated with 250-μl 0.25-M sodium carbonate, and placed at 95°C for 4 h. Cell suspensions were then adjusted to pH 5.2 with 1 M 150-μl acetic acid and 0.2 M 600-μl sodium acetate buffer (pH 5.2).

A 10-μl trehalase or 20-μL amylo-glycosidase (solubilized in 0.2 M sodium acetate pH 5.2) was added to cell suspensions for trehalose or glycogen determination, respectively. The intracellular glucose release was then quantified upon oxidase/peroxidase reagent treatment of the cells, using the glucose oxidase/peroxidase method (Cramp, 1967). Absorbance of the samples was measured, as described previously (Küçükgöze et al., 2013).

#### Catalase Activity Measurement

Catalase activity was assayed as described previously (Ueda et al., 1990). Briefly, 680-μl of 50-mM potassium phosphate buffer (pH 7.2) and 480-μl of 40-mM H_2_O_2_ were mixed and incubated in a quartz cuvette (2.5 min, 30°C). After incubation, 40-μl cell extract was added into this mixture to initiate the reaction. The decrease in absorbance at 240 nm was recorded and ΔA240/min/mg protein was calculated to determine the specific catalase activity.

#### Lyticase Sensitivity Assay

Lyticase sensitivity assay was adapted from Kuranda et al. (2006) and performed as described previously (Balaban et al., 2020), using the reference and the evolved strain cultured both under control and 0.5-mM H_2_O_2_ stress conditions. The cultures were then harvested (10 min; 10,000 g) and resuspended in 10-mL of 10-mM Tris/HCl buffer (pH 7.4), containing 40 mM β-mercaptoethanol (Merck, Hohenbrunn, Germany). Following incubation at 25°C for 30 min, 2 U/mL lyticase (Sigma-Aldrich, St. Louis, MO, United States) was added into each sample and then incubated at 30°C and 150 rpm. The lyticase resistance of the cells was calculated as the ratio of the OD_600_ value upon lyticase treatment to the initial OD_600_ value before lyticase treatment. Experiments were performed as three biological repeats.

### Transcriptomic Analysis by Microarray

Overnight cultures of the reference strain and the evolved strain were inoculated into fresh 2% YMM (0 mM and 0.5 mM H_2_O_2_) in 100 mL flasks to an initial OD_600_ of 0.1 and incubated at 30°C. At mid-exponential phase of growth, 1 OD_600_ unit of cells (2 × 10^7^ cells/mL) were harvested by centrifugation (Arslan et al., 2018a). Total RNA isolation of the harvested cultures was performed using RNeasy Mini Kit (Qiagen, Hilden, Germany), according to the manufacturer’s instructions. The experiment was repeated three times.

The RNA concentrations and quality were assessed by a NanoDrop 2000 UV-Vis spectrophotometer (Thermo Fisher Scientific, Waltham, MA, United States) and BioAnalyzer 2100 (Agilent Technologies, Santa Clara, CA, United States).

Agilent yeast microarrays, Yeast (V2) Gene Expression Microarray (Design ID:016322), were used to monitor mRNA transcript of *S. cerevisiae* open reading frames. Hybridization, washing, staining and scanning were performed according to the instructions in the Agilent One Color RNA Spike-in Kit manual. Gene expression profile was visualized, clustered hierarchically and expression fold changes of the evolved strain with respect to the reference strain were calculated using the Agilent GeneSpring GX software supplied by Agilent Technologies, Santa Clara, CA, United States as described previously (Arslan et al., 2018a).

KEGG pathways for stress tolerance were identified using DAVID Bioinformatics Resources (v6.8) (Huang et al., 2009), and the differentially expressed genes (*p* ≤ 0.05 and fold change ≥ 2) were determined, as described previously (Hacısalihoğlu et al., 2019). GO analysis (*p* ≤ 0.05 and fold change ≥ 2) was performed using Saccharomyces Genome Database SGD Gene Ontology Slim Mapper.^1^

The complete microarray data are available at GEO repository under accession number GSE184952.^2^

### Whole Genome Sequencing

Prior to sequencing, the reference strain and the evolved strain were grown and harvested as described before (Surmeli et al., 2019). DNA extraction was carried out using a MasterPure™ DNA Purification Kit (Epicenter, San Diego, United States), following the manufacturer’s instructions. Concentration and purity of isolated DNA samples was assessed by NanoDrop 2000 UV-Vis spectrophotometer (Thermo Fisher Scientific, Waltham, MA, United States) and Qubit^®^ Fluorometer 3.0 (Thermo Fisher Scientific).

The Ion Torrent Sequencing Systems were used for the whole genome sequencing of the strains. Library preparation involved four main phases: (i) Digestion and fragmentation of genomic DNA by using Ion Shear™ Plus Reagents Kit (Thermo Scientific, United States), (ii) ligation of adapters *via* use of IonXpress™ Plus Fragment Library Kit (Thermo Scientific, United States) and Ion Xpress™ Barcode Adapters, (iii) size selection with usage of E-Gel System (Thermo Scientific, United States and (iv) amplification and purification by using Ion Xpress™ Plus Fragment Library Kit (Thermo Scientific, United States) and Agencourt AMPure XP (Beckman Coulter, Carlsbad, CA, United States) reagent. All steps were carried out according to the manufacturer’s instructions.

Template preparation was carried out by the Ion Chef™ Instrument and high-throughput sequencing was performed by the Ion S5 Sequencing System (Thermo Scientific, United States). Briefly, 20 ng/mL of the prepared library was then loaded into Ion Chef™ (Thermo Fisher Scientific, Waltham, MA, United States). Ion 540™ Chip Kit was used for sample tracking and sequencing, using Ion S5™ Sequencer (Thermo Fisher Scientific, Waltham, MA, United States). Whole genome sequencing data were created and analyzed on Torrent Suite Software by using *S. cerevisiae* CEN.PK113-7D reference genome (GCA_000269885.1; ASM26988v1). The Torrent Suite Software was then used to perform raw data analysis at all stages including quality control, trimming adaptor, removing low-quality sequences, alignment of raw data to reference strain data and variant calling. Data from this work have been deposited in the NCBI Sequence Read Archive (SRA) under BioProject PRJNA768245.

### Statistical Analysis

All experiments were performed using at least three biological replicates. The data analyses were performed using the R software “stats” package (R Core Team, 2020). Statistical significance was calculated using a two-tailed, unpaired Student’s *t*-test. *p* < 0.05 was considered as statistically significant except for analyses of microarray and whole genome sequencing.

## Results

### Identification of the Evolved Strains With the Highest Oxidative Stress Resistance Within the Final Populations of Diverse Evolutionary Selection Strategies

Evolutionary engineering strategies consisted of successive batch selection in the presence of gradually increasing continuous oxidative stress with and without pre-treatments. The starting oxidative stress level was set as 0.5 mM H_2_O_2._ As the growth of the 21st–24th populations was as low as 0.1–0.4 OD_600_, the selection experiments to obtain oxidative stress-resistant evolved strains ended with these final populations.

**FIGURE 2:**
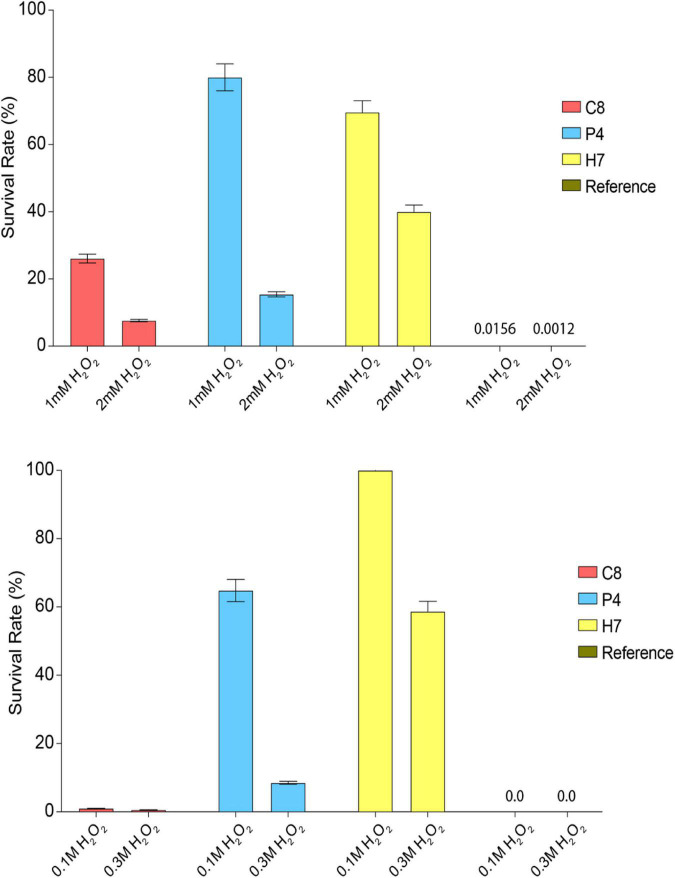
Oxidative stress survival rates of the reference and most resistant strains C8, P4, and H7 obtained from OC, OP, and HP selection approaches, respectively. The cultures were **(top figure)** continuously exposed to 1 or 2 mM H_2_O_2_ or **(bottom figure)** exposed to 0.1 and 0.3 M H_2_O_2_ as pulse oxidative stress. The survival rates (%) were determined upon incubation (72 h, 30^°^C).

The oxidative stress resistance levels of individual evolved strains isolated from the final populations of these different evolutionary engineering strategies were then determined. Three evolved strains called C8 (OC strategy), P4 (OP strategy), and H7 (HP strategy) were identified as the highest oxidative stress-resistant strains, upon continuous or pulse oxidative stress tests, using the MPN method. The genetic stability of the evolved strains C8, P4, and H7 was verified by 10 successive batch cultivations in the absence of oxidative stress as the selection pressure (data not shown). The survival rates of the evolved strains H7 and P4 were higher than those of the reference strain and C8, under both continuous and pulse oxidative stress conditions, despite a relatively better survival of C8 under continuous stress condition, compared to the pulse stress condition. Besides, H7 had the highest survival rate under most of the stress conditions tested, except for 1 mM continuous H_2_O_2_ stress (Figure 2).

### Cross-Resistance to Other Stress Factors

**FIGURE 3:**
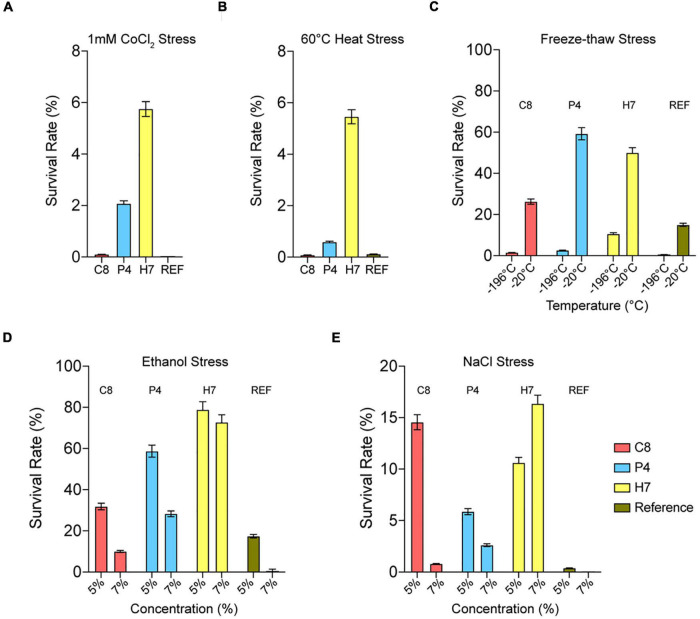
Quantitative cross-resistance analysis (MPN) results of the highest oxidative-stress resistant strains C8, P4, H7 and the reference strain. The cultures were exposed to **(A)** 1 mM CoCl_2_, **(B)** 60^°^C for 10 min, **(C)** –196^°^C (liquid nitrogen) and –20^°^C for 25 min, **(D)** 5 and 7% (v/v) ethanol and **(E)** 5 and 7% (w/v) NaCl. The survival rates (%) were determined upon incubation (72 h, 30^°^C). The results were obtained using the MPN analysis under various stress conditions.

Cross-resistance of evolved strains C8, P4, and H7 to different stress types including cobalt (1 mM CoCl_2_), heat (60°C for 10 min), freeze-thaw [–196°C (liquid nitrogen) and –20°C for 25 min], ethanol [5 and 7% (v/v)], and salt [5 and 7% (w/v) NaCl] stress were also tested (Figure 3).

The results showed that the evolved strain H7 had the highest cross-resistance to cobalt and heat stress, compared to C8 and P4 (Figures 3A,B). H7 was also cross-resistant to freeze-thaw stress (Figure 3C), and highly cross-resistant to ethanol stress (5 and 7% “v/v”) (Figure 3D). Similar results were also observed for salt stress-resistance (5 and 7% “w/v” NaCl) (Figure 3E).

**FIGURE 4:**
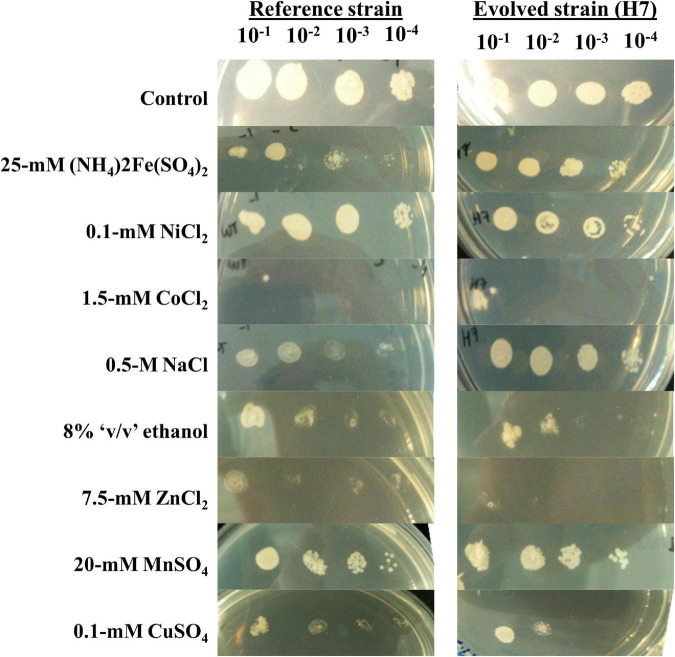
Semi-quantitative cross-resistance analysis (spot assay) results of H7 and the reference strain, upon 72 h of growth. The strains were cultured on YMM plates containing various stress factors, at four serial dilutions (10^– 1^ to 10^– 4^). YMM plates without any stress factor were used as the control.

Semi-quantitative cross-resistance test results of the evolved strain H7 were also generally in line with the quantitative, MPN-based cross-resistance results. Additionally, H7 was cross-resistant to iron [25 mM (NH_4_)2Fe(SO_4_)_2_] as shown by the semi-quantitative cross-resistance test results (Figure 4).

### Physiological and Metabolic Characterization of the Evolved Strain

**FIGURE 5:**
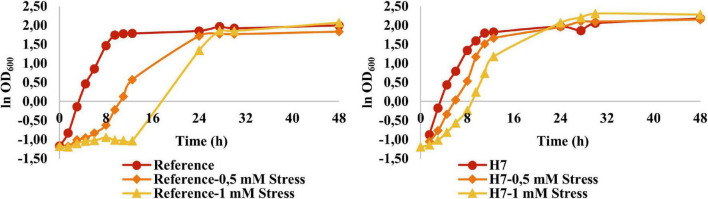
Growth behavior of the reference strain and H7 in the absence and presence of 0.5-mM and 1-mM H_2_O_2_ as the oxidative stress factor. The cultures were grown in YMM at 30^–^C and 150 rpm for 48 h.

Growth physiology of the reference (*905*) and the evolved strain (H7) was analyzed using batch cultures in YMM and YMM containing 0.5-mM and 1-mM H_2_O_2_ as the oxidative stress factor (Figure 5).

The maximum specific growth rates and doubling times of the cultures are indicated in Table 1.

**TABLE 1:** Maximum specific growth rates (μ_max_ “h^–1^”) and doubling times (h) of the reference strain (905) and the evolved strain (H7) in the absence and presence of 0.5-mM and 1-mM H_2_O_2_ stress.

	μ_max_ (h^–1^)	Doubling time (h)
*905*	0.35	2.00
*905* 0.5-mM stress	0.24	2.92
*905* 1-mM stress	0.15	4.77
H7	0.33	2.12
H7 0.5-mM stress	0.28	2.47
H7 1-mM stress	0.30	2.32

It was observed that the oxidative stress levels (up to 1 mM H_2_O_2_) applied in these experiments had no significant inhibitory effect on the growth of H7. However, the reference strain was significantly inhibited at these oxidative stress levels. At 1 mM H_2_O_2_ in particular, the reference strain had a considerably longer lag phase and doubling time, as well as a significantly lower maximum specific growth rate (Figure 5 and Table 1).

**FIGURE 6:**
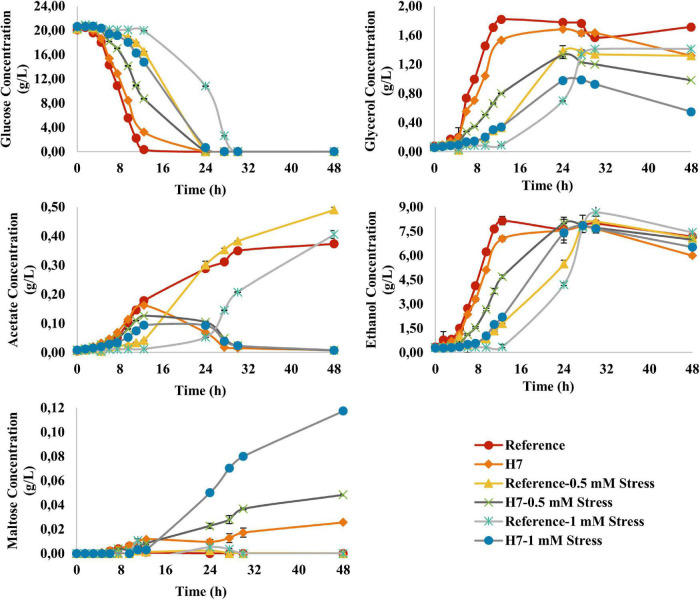
Residual glucose concentration, glycerol, ethanol, acetate and maltose production (g L^– 1^) profiles of the reference strain and H7 in the absence and presence of 0.5-mM and 1-mM H_2_O_2_ as the oxidative stress factor. The cultures were grown in YMM at 30^°^C and 150 rpm for 48 h.

The metabolite profiles of both strains were generally in line with their growth data (Figure 6). The reference strain had a significantly decreased glucose consumption rate at 1 mM H_2_O_2_, whereas the glucose consumption rate of H7 did not significantly decrease during oxidative stress. Glycerol production (g/L) profiles of the reference strain *905* and H7 cultures varied in line with their growth curves. Interestingly, it was observed that under oxidative stress conditions, the glycerol levels of H7 slightly decreased after the first 24 h of cultivation, during the stationary phase of growth. Acetate was produced by both reference strain and H7, although at significantly lower levels in H7, compared to the reference strain. Additionally, after the exponential phase of growth, acetate levels decreased in H7 cultures, both in the presence and absence of oxidative stress. In the presence of oxidative stress, ethanol production of H7 was higher than that of the reference strain. François and Parrou (2001) reported that, under harsh environmental conditions, glycogen production and degradation occurs in yeast which leads to the liberation and accumulation of maltose. For this reason, maltose levels of H7 and the reference strain were also measured. Interestingly, H7 accumulated maltose, unlike the reference strain. As the H_2_O_2_ concentration (oxidative stress level) in the culture medium increased, the maltose production levels of H7 also increased (Figure 6).

**FIGURE 7:**
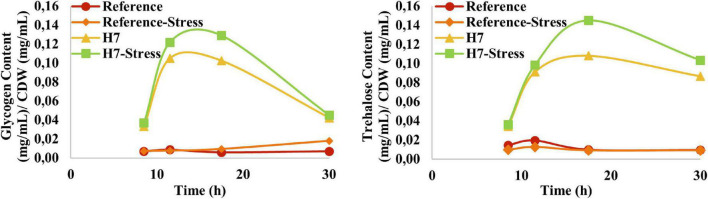
Glycogen and trehalose production (mg mL^−1^) per CDW (mg mL^−1^) profiles of the reference strain and H7 in the absence and presence of 0.5-mM H_2_O_2_ as the oxidative stress factor. The cultures were grown at 30^°^C and 150 rpm, using YMM. Glycogen and trehalose contents of the samples were determined enzymatically, between the 10th and 30th hour of cultivation.

As glycogen and trehalose function both as storage carbohydrates and stress protectants (François and Parrou, 2001), the glycogen and trehalose profiles of the reference strain and the evolved strain H7 were obtained (Figure 7). The results revealed that H7 had significantly higher glycogen and trehalose content than the reference strain throughout the cultivation, both in the presence and absence of oxidative stress. Both glycogen and trehalose contents of H7 increased until the end of the exponential phase of growth (approximately at the 18th hour of cultivation), after which it decreased. The maximum glycogen and trehalose contents of H7 at the 18th hour of cultivation were 17-fold and 10-fold higher than those of the reference strain (Figure 7).

**FIGURE 8:**
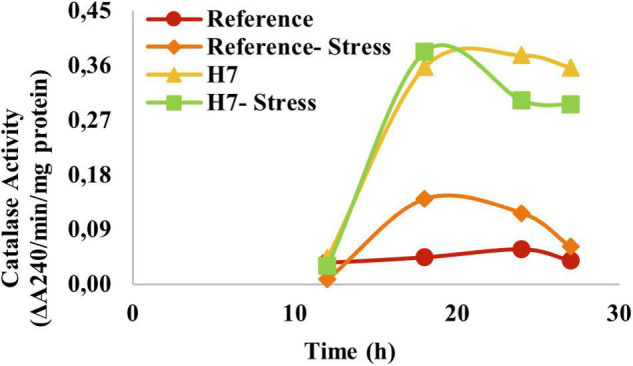
Specific catalase activity (ΔA_240_/min/mg protein) of the reference strain and H7 in the absence and presence of 2-mM H_2_O_2_ as the oxidative stress factor. The cultures were grown in YMM at 30^°^C and 150 rpm.

To test if the oxidative stress-resistant evolved strain also had increased catalase activity, the catalase activity measurements were made for the evolved strain and the reference strain. It was observed that the specific catalase activity of H7 was about fourfold of that of the reference strain, both in the presence and absence of oxidative stress (Figure 8), without induction of catalase activity by H_2_O_2_ in H7.

**FIGURE 9:**
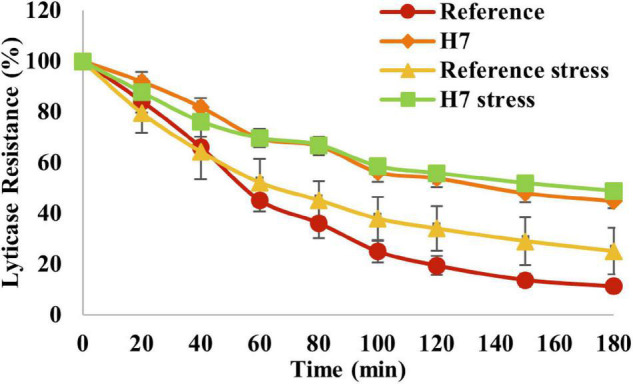
Lyticase sensitivity assay results of the evolved strain H7 and the reference strain under oxidative stress (0.5-mM H_2_O_2_) and control conditions. Lyticase sensitivity was calculated as the percent decrease in lyticase resistance from 100% as the initial value.

The lyticase sensitivity assay was also applied to test if there were differences in cell wall integrity between the evolved strain and the reference strain. The results revealed that H7 had significantly higher lyticase resistance than the reference strain, both in the presence and absence of oxidative stress (Figure 9), indicating a significantly higher cell wall integrity or robustness of H7. Interestingly, the lyticase resistance of H7 did not increase in the presence of oxidative stress, although the lyticase resistance of the reference strain slightly increased upon oxidative stress (Figure 9).

### Comparative Gene Expression Profile of the Oxidative Stress-Resistant Evolved Strain H7

To investigate the molecular basis of oxidative stress resistance, global transcriptomic profiles of H7 and the reference strain were analyzed during mid-exponential growth phase and in the absence of oxidative stress, by using DNA microarray technology. *p* < 0.05 was applied as the cut-off value. 1911 ORFs were differentially expressed by at least two-fold in the evolved strain. Of these, 897 were up-regulated and 1014 were down-regulated. Genes of H7 which were up-regulated by sevenfold or higher and down-regulated by at least fivefold are shown in Tables 2, 3 respectively. Gene ontology analysis was carried out by GO Slimmapper of the Saccharomyces Genome Database. Genes that were up- and down-regulated by at least twofold in H7 are listed in Supplementary Tables 1, 2, respectively.

**TABLE 2:** Genes which were up-regulated by at least sevenfold in H7.

Systematic gene symbol	Standard gene symbol	Gene name	Fold change	Regulatory genes that are known to regulate the stated gene
*YMR107W*	*SPG4*	Stationary phase gene	9.52	*CST6, FKH1, GCR1, LEU3, MED2, RAP1, SPT10, SRB5, UME6, YAP5*
*YEL039C*	*CYC7*	CYtochrome C	9.09	*TUP1, FKH1, FKH2, HAP1, HIR3, MSN2, PHD1, SFP1, SIR2, SIR3, SIR4, SUA7, TFC7*
*YMR206W*		Putative protein of unknown function	8.44	*GCR2, SFP1, SIR2, SIR3, SIR4, SUA7, UGA3, UME6, XBP1*
*YFL014W*	*HSP12*	Heat shock protein	8.44	*CLA4, BAS1, CBF1, CSE2, CST6, GCN5, GCR1, HAL9, HCM1, HSF1, IXR1, MED4, MSN2, MSN4, NCB2, RAP1, REB1, RPN4, SFP1, SIR2, SIR3, SIR4, SPT10, SPT3, SRB8, STE20, SUA7, TUP1, UME6, XBP1, YAP5, YAP6, ZAP1*
*YNL194C*		Integral membrane protein, similar to *SUR7*	8.30	*AFT2, CYC8, MSN2, MSN4, NCB2, RPD3, SPT3, SUA7, UME6, YAP5*
*YDL222C*	*FMP45*	Found in mitochondrial proteome	7.89	*BUR6, MED4, STE12, SUA7, TEC1, XBP1*
*YIL057C*	*RGI2*	Respiratory growth induced	7.69	*FKH2, MSN2, MSN4, RAP1, SPT10*
*YFR053C*	*HXK1*	HeXoKinase	7.63	*BUR6, CYC8, GCN4, GCN5, HSF1, IXR1, MED2, MED4, MSN2, MSN4, RAP1, SFP1, SIN4, SPN1, SPT6, STP1, SUA7, TUP1, YAP1*
*YBR072W*	*HSP26*	Heat shock protein	7.32	*HCM1, SNF2, BUR6, CIN5, CST6, CYC8, FKH2, GCN5, HSF1, IFH1, MED2, MED4, MET32, MET4, MSN2, MSN4, NCB2, NHP6A, PHO2, RAP1, REB1, SFP1, SPN1, SPT10, SPT20, SPT3, SPT7, SRB5, SUA7, TOA2, TUP1, XBP1, ZAP1*
*YNL196C*	*SLZ1*	Sporulation-specific protein with a leucine zipper motif	7.27	*SPT10*
*YLR327C*	*TMA10*	Translation machinery associated	7.23	*BUR6, CAD1, CST6, CUP2, CYC8, FKH1, FKH2, FLO8, GCR1, HSF1, MED2, MED4, MET32, MET4, MSN2, MSN4, NCB2, RAP1, RGM1, RDR1, SFP1, SPT10, SPT3, SPT7, SRB5, SUA7S, WI4, SWI6, TUP1, YAP1*
*YAL061W*	*BDH2*	Putative medium-chain alcohol dehydrogenase	7.15	*BUR6, CST6, CYC8, MET32, MET4, SOK2, SPT10, SUA7, YAP1*
*YBR285W*		Putative protein of unknown function	7.13	*FKH1, GCN5, LEU3, RPD3, SFP1, SPT10, SUA7, UME6, ZAP1*
*YOL052C-A*	*DDR2*	DNA damage responsive	7.00	*CBF1, CST6, GCR1, HSF1, IXR1, LEU3, MET32, MET4, MSN2, MSN4, RAP1, REB1, SIR2, SIR3, SIR4, SPT10, SRB8, SSN2, SUA7*

**TABLE 3:** Genes that were down-regulated by at least fivefold in H7.

Systematic gene symbol	Standard gene symbol	Gene name	Fold change	Regulatory genes that are known to regulate the stated gene
*YJL167W*	*ERG20*	ERGosterol biosynthesis	7.00	*CBF1, INO4, RAP1, REB1, SFP1*
*YDR035W*	*ARO3*	AROmatic amino acid requiring	6.45	*FKH1, FKH2, GCN4, SPT6, UME6*
*Q0110*	*BI2*	Cytochrome b mRNA maturase bI2	6.36	
*YGR245C*	*SDA1*	Severe depolymerization of actin	5.70	*SPF1*
*YGL255W*	*ZRT1*	Zinc-regulated transporter	5.64	*MOT3, ROX1, ADA2, FKH1, GCN4, GZF3, IXR1, MOT3, RFX1, RIM101, ROX1, SFP1, SIN3, SIR2, SIR3, SIR4, SRB8, SSN2, STP1, SWI4, UME1, XBP1, YAP6, ZAP1*
*YGL256W*	*ADH4*	Alcohol DeHydrogenase	5.36	*FKH1, FKH2, SFP1, SPT10, TYE7, YAP5, ZAP1*
*YNL112W*	*DBP2*	Dead box protein	5.24	*CDC73, FKH1, FKH2, RFX1, SFP1, ZAP1*
*YAR075W*		Non-functional protein with homology IMP dehydrogenase	4.48	*CST6, GCR1, HSF1, RAP1, SFP1, SFP1, SIR2, SIR3, SIR4, SPT10, XBP1, YAP1, YAP6*

Table 2 and Supplementary Table 3, show many of the up-regulated genes are associated with the response to stress conditions such as oxidative, heat, chemical, osmotic, and starvation stress. In addition, genes related to metabolite production and modification process such as the carbohydrate metabolic process, generation of precursor metabolites and energy, the monocarboxylic acid metabolic process, the cofactor metabolic process, the lipid metabolic process, protein phosphorylation, protein complex biogenesis, cell wall organization or biogenesis, oligosaccharide metabolic process, and protein modification by small protein conjugation or removal and protein folding were also up-regulated in H7. Mitochondrion and peroxisome organization-related genes were also up-regulated. Transport-related genes such as ion transport, transmembrane transport, protein targeting, carbohydrate transport, Golgi vesicle transport, membrane invagination, membrane fusion, lipid transport, amino acid transport and organelle fusion were also generally up-regulated, excluding nuclear transportation. Further, genes related to cellular respiration were also up-regulated. Interestingly, the meiotic cell cycle and sporulation-related genes were also overexpressed. Table 3 and Supplementary Table 2 indicate that a major group of down-regulated genes in H7 belongs to ribosome biosynthesis. The down-regulated genes also have a role in RNA biosynthesis and modification processing, such as tRNA biosynthesis, snoRNA processing, RNA modification, translation and RNA splicing. A considerable number of genes down-regulated play a role in processes related to nucleotide metabolism such as production, assembly and transport. Genes associated with nuclear transport and transport of nucleobase-containing metabolites were mostly down-regulated in H7. Transcription of genes for organelle and ribosome assembly processes were also down-regulated. Other down-regulated genes in H7 were related to mitosis and cell cycle. Conversely, sporulation and stationary phase-related genes were strongly up-regulated in H7.

As shown in Supplementary Table 3, genes related to stress response; metabolic processes of carbohydrates, lipids, and proteins including anabolic and catabolic reactions; generation of precursor metabolites and energy; and transportation of metabolites and ions were up-regulated in H7. However, expression levels of the genes that belong to processes related to ribosome, RNA, nuclear transport, organelle assembly, tRNA, cell cycle, mitosis and transcription from RNA polymerases decreased in H7.

**FIGURE 10:**
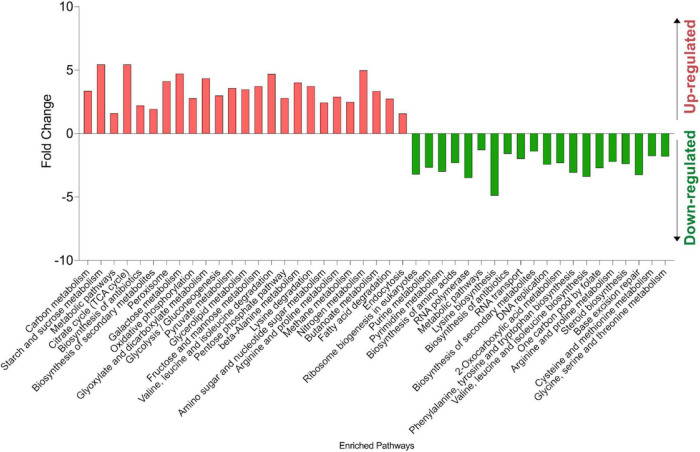
KEGG pathway analysis results of up- and down-regulated genes in the oxidative stress-resistant evolved strain H7, compared to the reference strain. The experiments were performed as three biological replicates.

A KEGG pathway mapping analysis was performed for the up- and down-regulated gene sets using DAVID v6.8 Bioinformatics Resources (Huang et al., 2008, 2009) to assess their biological significance. The analysis identified nucleobase metabolism, RNA polymerase and ribosome biogenesis, amino acids biosynthesis and steroid biosynthesis as significantly enriched pathways in the down-regulated gene sets. Moreover, pathway analysis showed that the up-regulated genes in H7 were enriched primarily with those associated with carbon metabolism, oxidative phosphorylation, peroxisome, fatty acid degradation and endocytosis pathways (Figure 10).

### Single Nucleotide Variation Analysis

Variant analysis was carried out for more than 20 million reads for both strains with an average coverage depth of 350. Coverage and quality data are given in Supplementary Table 4. We have detected 33 missense, 2 nonsense, and 1 frameshift deletion mutations in the evolved strain H7. Further, 12 silent mutations, 12 intergenic mutations, and 1 nucleotide substitution on Long Terminal Repeat were observed (Table 4).

**TABLE 4:** Mutation positions and types in the genome of the oxidative stress-resistant evolved strain H7.

Gene name	Genetic change	Mutation positions	Description (Cherry et al., 2012)
*AIM19*	c.154G > A	p.P52S	Protein of unknown function; mitochondrial protein that physically interacts with Tim23p
*ATG40*	c.-602G > A		Autophagy receptor with a role in endoplasmic reticulum degradation
*Between RPS14A and snR189*	III:177852 G > A		
*CDC25*	c.1601T > A	p.I534K	Membrane bound guanine nucleotide exchange factor; also known as a GEF or GDP-release factor
*CFT2*	c.1945G > A	p.G145S	Subunit of the mRNA cleavage and polyadenylation factor (CPF); required for pre-mRNA cleavage,
*CIP1*	c.1010G > A	p.R337K	Cyclin-dependent kinase inhibitor; activated by environmental stress
*DUF1*	c.2164G > A	p.Q722*	Ubiquitin-binding protein of unknown function; contains one WD40 repeat in a beta-propeller fold
*DYN1*	c.10724C > T	p.G3575E	Cytoplasmic heavy chain dynein; involved in spindle assembly, chromosome movement, and spindle orientation during cell division, targeted to microtubule tips by Pac1p
*FIS1*	c.193G > A	p.L65F	Protein involved in mitochondrial fission and peroxisome abundance; may have a distinct role in tethering protein aggregates to mitochondria in order to retain them in the mother cell
*FTH1*	c.211delC	p.Q71K^#^	Putative high affinity iron transporter; involved in transport of intravacuolar stores of iron
*GDH2*	c.1880C > T	p.G627D	NAD(+)-dependent glutamate dehydrogenase; degrades glutamate to ammonia and alpha-ketoglutarate
*HAT2*	c.892G > A	p.V298M	Subunit of the Hat1p-Hat2p histone acetyltransferase complex; required for high affinity binding of the complex to free histone H4, thereby enhancing Hat1p activity
*HOM3*	c.800G > A	p.P267L	Aspartate kinase (L-aspartate 4-P-transferase); cytoplasmic enzyme that catalyzes the first step in the common pathway for methionine and threonine biosynthesis
*HPT1*	c.179G > A	p.R60K	Dimeric hypoxanthine-guanine phosphoribosyltransferase
*IES1*	c.307C > T	p.G103R	Subunit of the INO80 chromatin remodeling complex
*IKS1*	c.554C > T	p.G185D	Protein kinase of unknown cellular role; putative serine/threonine kinase
*IRC15*	c.217G > A	p.L73F	Microtubule associated protein; regulates microtubule dynamics; required for accurate meiotic chromosome segregation
*LYS14*	c.2373 + 53 C > T		Transcriptional activator involved in regulating lysine biosynthesis; involved in the regulation of genes of the lysine biosynthesis pathway
*MAK10*	c.1906G > A	p.R636K	Non-catalytic subunit of the NatC N-terminal acetyltransferase
*MCD4*	c.2760 + 918C > T		Protein involved in GPI anchor synthesis; multimembrane-spanning protein that localizes to the endoplasmic reticulum;
*MEO1*	c.11C > T	p.T4I	Putative protein of unknown function;
*MSC3*	c.2187 + 234C > T		Protein of unknown function
*MSH1*	c.2880 + 69C > T		DNA-binding protein of the mitochondria; involved in repair of mitochondrial DNA
*NFT1*	c.2186C > T	p.T729M	Putative transporter of the MRP subfamily
*NOP9*	c.1939A > C	p.S647A	Essential subunit of U3-containing 90S preribosome; involved in production of 18S rRNA and assembly of small ribosomal subunit
*NRG1*	c.245C > T	p.W82*	Transcriptional repressor; mediates glucose repression and negatively regulates a variety of processes including filamentous growth and alkaline pH response
*PIG1*	c.1892G > T	p.S631N	Putative targeting subunit for type-1 protein phosphatase Glc7p
*PPX1*	c.567C > T	p.M189I	Exopolyphosphatase; hydrolyzes inorganic polyphosphate (poly P) into Pi residues
*PRP16*	c.251C > T	p.A84V	DEAH-box RNA helicase involved in second catalytic step of splicing and in exon ligation
*PRP5*	c.2147A > T	p.K716M	RNA helicase in the DEAD-box family; necessary for prespliceosome formation
*RER1*	c.567 + 47G > A		Protein involved in retention of membrane proteins
*RPF2*	c.433C > T	p.G145S	Essential protein involved in rRNA maturation and ribosomal assembly; involved in the processing of pre-rRNA and the assembly of the 60S ribosomal subunit
*RRP6*	c.1720A > G	p.K574E	Nuclear exosome exonuclease component; involved in RNA processing, maturation, surveillance, degradation, tethering, and export; role in sn/snoRNAs precursor degradation
*RSC30*	c.1402G > A	p. E68K	Component of the RSC chromatin remodeling complex
*RTT10*	c.2498G > A	p.S833L	WD40 domain-containing protein involved in endosomal recycling
*SDC25 (CDC25p)*	c.2204G > A	p.G735D	Pseudogene; localized to the membrane; expressed in poor nutrient conditions and on non-fermentable carbon sources
*SPC105*	c.83G > A	p.S28N	Subunit of a kinetochore-microtubule binding complex
*SPP41*	c.2769G > A	p.M923I	Protein of unknown function
*UFD1*	c.634G > A	p.A212T	Substrate-recruiting cofactor of the Cdc48p-Npl4p-Ufd1p segregase
*UFO1*	c.-591G > A		F-box receptor protein; binds to phosphorylated Ho endonuclease, allowing its ubiquitination by SCF and subsequent degradation
*VBA1*	c.-31G > A		Permease of basic amino acids in the vacuolar membrane
*VTC1*	c.248G > A	p.R83K	Regulatory subunit of the vacuolar transporter chaperone (VTC) complex; VTC complex is involved in membrane trafficking, vacuolar polyphosphate accumulation, microautophagy and non-autophagic vacuolar fusion
*YCR101C*	c.250C > T	p.P84S	Putative protein of unknown function
*YGL039W*	c.-364 G > A		Aldehyde reductase; shown to reduce carbonyl compounds to chiral alcohols
*YGL052W*	c. 112G > A	p.A38T	Dubious open reading frame
*YHRWdelta13*	c.211C > T		Ty1 LTR
*YJL127C-B*	c.159 + 19		Mitochondrial protein of unknown function
*YMR321C*	c. 318 + 747G > A		Putative protein of unknown function
*YPS1*	c.838G > A	p.P280S	Aspartic protease; involved with other yapsins in the cell wall integrity response

^#^Frameshift mutation. *Nonsense mutation.

## Discussion

In this study, we particularly focused on oxidative stress, one of the most common stress factors in industrial applications using yeast. We investigated the genetic and molecular mechanisms that provide yeast cells an advantage over previous generations to cope with this stress condition (Gasch et al., 2000; Alexandre et al., 2001; Morano et al., 2012). We first constructed oxidative stress-resistant and genetically stable evolved strains, using an evolutionary engineering approach under oxidative stress conditions, with or without heat pretreatment and oxidative stress (hydrogen peroxide) pretreatment. We then performed comparative genomic, transcriptomic, and physiological analyses with the most efficient oxidative stress-resistant evolved strain (H7) and the reference strain, to elucidate the molecular resistance mechanisms to this stress condition.

The results revealed that the evolutionary engineering strategy under oxidative stress conditions and heat pretreatment yielded a genetically stable, evolved strain (H7) with the highest oxidative stress resistance level and with cross-resistance to many other stress types including cobalt, freeze-thaw, heat, ethanol, iron, and salt stress (Figures 2, 3). This evolutionary advantage may be associated with gaining thermotolerance *via* heat shock factors and stress response element pathways that regulate the synthesis of heat shock proteins (Hsps) and with increasing the robustness of the cells to withstand the damaging effects of oxidative stress (Morano et al., 1998; Verghese et al., 2012; Rikhvanov et al., 2014). Indeed, many *HSP* genes were upregulated in our evolved strain H7 (Supplementary Table 1) and 11 upregulated genes were found in the “response to heat” category for H7 (Supplementary Table 3). Many Hsps can be induced in cells recovering from mild heat shock; they are responsible for the refolding of heat-denatured proteins and survival which can help survive under various stress conditions (Seppä and Makarow, 2005). In line with this notion, an early adaptive response provides almost immediate protection against sublethal stress conditions by activating preexisting defenses; while a late adaptive response provides more efficient protection against a severe stress and also allows cells to return to non-stress conditions, as reported previously (Costa and Moradas-Ferreira, 2001).

The maximum specific growth rate/doubling time of H7 were unaffected by oxidative stress conditions, and the catalase activity of H7 was higher than the reference strain even in the absence of H_2_O_2_ stress. This strongly suggests the effective resistance of H7 to oxidative stress. Among the many environmental stress response genes that were upregulated in the evolved strain H7, *CTT1* that encodes cytosolic catalase T was up-regulated by 5.37-fold in H7, compared to the reference strain. Cytosolic catalase T is known to have a role in protection from oxidative damage caused by H_2_O_2_ (Wieser et al., 1991). Another adaptation that is most likely associated with heat pretreatment dependent adaptation was observed in the stress-regulatory transcription factors Msn2 and Msn4. Many genes that are regulated by Msn2 and Msn4 were highly up-regulated in H7 (Table 2). These transcription factors, as alternatives to *CTT1*, activate expression of the genes involved in fatty acid oxidation. Even in their absence, *CTT1* can take control over the entire resistance mechanism machinery against oxidative stress (Hasan et al., 2002; Rajvanshi et al., 2017). In addition, physiological analyses showed that, unlike the reference strain, the concentrations of glycerol produced by H7 decreased during the stationary phase of growth, possibly indicating glycerol consumption by H7 (Figure 6). Transcriptomic data also supports the concept of elevated glycerol utilization in the evolved strain H7 for energy production *via* upregulation of *GUT1* (Glycerol kinase, involved in glycerol utilization) (Pavlik et al., 1993), *GUT2* (responsible for binding to the inner mitochondrial membrane glycerol-3-phosphate dehydrogenase) (Rønnow and Kielland-Brandt, 1993), *GCY1* (glycerol dehydrogenase, plays a role in an alternative pathway for glycerol catabolism under microaerobic conditions) (Jung et al., 2012), and *DAK1* (Dihydroxyacetone kinase, involved in stress adaptation by using dihydroxyacetone as the carbon and energy source) (Molin et al., 2003). Meanwhile, trehalose-associated genes *TPS1, TPS2*, and *TSL1* were up-regulated by 2.4, 3.0, and 5.4-fold in H7, compared to the reference strain, respectively (Supplementary Table 1). These findings also support the significantly higher trehalose levels observed in H7 (Figure 7). Changes in gene expression profile related to glycerol metabolism and trehalose production could also be associated with protecting yeast against oxidative stress which warrants further investigation. In addition, the potential relationship between oxidative stress resistance and acetate levels is yet to be clarified. Acetate levels of H7 decreased after the exponential phase of growth, both in the presence and absence of oxidative stress, which was not observed in the reference strain (Figure 6). It is known that unused acetate is released into the culture medium as acetic acid (Walkey et al., 2012). Up-regulation of *ADY2* (6.2-fold) and *JEN1* (6.1-fold) in H7 support this condition, as *ADY2* encodes an acetate transporter required for sporulation and Jen1p has a role in a lactate–pyruvate–acetate–propionate transport (Paiva et al., 2004). The strong up-regulation of these two genes in H7 may allow for a large amount of acetate uptake into the cell. Further, up-regulation of *ACS1* (1.7-fold) might indicate the utilization of acetate, as Acs1p has a role in acetate utilization and is required for growth on acetate under glucose starvation (Takahashi et al., 2006). The notable upregulation of the acetate transporter genes and the decrease in acetate concentrations may indicate that H7 might accumulate acetate inside the cell. Interestingly, a previous study showed that pre-incubation of yeast cells with low amounts of H_2_O_2_ caused protection against high acetate levels (Semchyshyn et al., 2011).

The decreased levels of glycerol and acetate during late phases of growth may also suggest that the evolved strain H7 might have adapted itself to grow on non-fermentable carbon sources. Our transcriptomic data also supports this, where *HXK1*, *GLK1*, and *MPC3* genes – that are known to be expressed during growth on non-fermentable carbon sources (Rodríguez et al., 2001; Cherry et al., 2012) – were up-regulated in H7 by 7.6, 3.9, and 6.8-fold, respectively. Another major physiological difference between the evolved strain H7 and the reference strain was the significantly higher maltose levels observed in H7, particularly during the late phases of growth (Figure 6). The higher maltose levels in H7 may be associated with the up-regulation of *SGA1* (by 4.8-fold), as *SGA1* encodes a protein involved in glycogen breakdown and the release of maltose (Pugh et al., 1989). It is known that glycogen metabolism is enhanced in response to a wide variety of environmental stresses, and glycogen degradation can lead to the liberation and accumulation of maltose (François and Parrou, 2001). The oxidative-stress-resistant evolved strain H7 may have such a profile, and it seems to activate stress response genes even in the absence of oxidative stress, retaining an alertness for any potential stress conditions. *HSP12*, *HSP26*, and *DDR2*, other important environmental stress response genes, were also significantly up-regulated in H7 compared to reference strain. In addition, like with glycogen concentrations, H7 trehalose levels were also high, both in the presence and absence of oxidative stress. Similar observations were made in our previous study with a cobalt-resistant *S. cerevisiae* strain, where the expression level of *COT1*, a gene encoding a major vacuolar cobalt-transporter, significantly increased in the cobalt-resistant strain, even in the absence of cobalt stress (Alkim et al., 2013).

We had shown in a previous chronological life span (CLS) study that H7 was a long-lived strain, compared to the reference strain (Arslan et al., 2018b). The link between oxidative stress-resistance, longevity and autophagy has been reported previously (Orozco et al., 2012). Thirty autophagy-related genes were up-regulated by more than twofold for H7. In particular, *ATG39* was increased 5.6-fold in H7. Atg39p is located in perinuclear ER (or the nuclear envelope) and induces autophagic sequestration in part of the nucleus. Atg39-dependent autophagy in the perinuclear ER/nucleus is required for cell survival under nitrogen-deprived conditions. Atg39p has a major role in perinuclear ER-phagy (Mochida et al., 2015). Although there was no change in expression level in the *ATG40* gene, its upstream mutation in H7 may have caused a significant change in autophagy. Besides the *ATG40* gene, *VTC1* gene of H7, which is vacuole-related, also has a missense mutation. *VTC1* encodes the regulatory subunit of the vacuolar transporter chaperone (VTC) complex which is an important constituent of autophagic tubes required for the scission of microautophagic vesicles from these tubes (Uttenweiler et al., 2006). In a previous study, we showed that an iron-stress-resistant evolved *S. cerevisiae* strain had mutations in *VBA2* (encodes a permease) and *VTC4* (encodes a VTC complex regulatory subunit) genes (Balaban et al., 2020). It is important to note that H7 and this iron-resistant evolved strain, which is also resistant to oxidative stress, have mutations on genes related to vacuolar functions. These mutations could be important for oxidative stress. The up-regulation of autophagy-related genes in H7 may enhance its autophagy activity, and mutations in vacuole-related genes may be useful to eliminate the degraded macromolecules *via* vacuoles.

One of the most important mutations in H7 is the nonsense mutation in the *NRG1* gene. Nrg1p is a transcriptional repressor encoded by *NRG1*. The nonsense mutation might lead to the synthesis of a truncated protein with a total or partial loss of its functionality. *NRG1* regulates 2.41% of the genes that were down-regulated by at least twofold, and 8.97% of the genes that were up-regulated by at least twofold in H7 (Supplementary Table 5). Nrg1p is a negative regulator of glucose-repressed genes and nrg1Δ mutants could utilize several different carbon sources (Zhou and Winston, 2001). It is important to note that Nrg1p controls the up-regulated genes in H7 that have a role in oxidative stress tolerance or that are induced by glucose limitation. Ten genes have been down-regulated by more than twofold in H7, and 33 genes have been up-regulated by more than twofold (Supplementary Table 5). Up-regulated genes regulated by *NRG1* play a role in oxidative stress tolerance or they are induced by a reaction to limited glucose availability – *CCP1* encodes mitochondrial cytochrome-c peroxidase, and it was up-regulated by 2.5-fold in H7 (Supplementary Tables 5, 6). Hydrogen peroxide exposure induces activation of the mitochondrial cytochrome-c peroxidase (Charizanis et al., 1999). *GSY1* encodes glycogen synthase (Farkas et al., 1990), and it was also up-regulated in H7 by 4.8-fold. *HXT2* encodes a high affinity, low-capacity glucose transporter and is induced and expressed under low-glucose conditions (Johnston and Kim, 2005). *HXT2* was up-regulated by 4.8-fold in H7. In addition to the genes that have a role in metabolic maintenance, genes encoding transcription factors were also up-regulated in H7, such as *HAP4*, *NRG2*, and *USV1* which are targets of *NRG1*.

A missense mutation was found in H7 on the *RSC30* gene which encodes a regulatory protein according to the YEASTRACT database (Teixeira et al., 2018). In H7, this gene regulates 5.32% of the down-regulated and 8.18% of up-regulated genes with a fold change higher than 2. *RSC30* is required for the proper regulation of cell wall/stress response and ribosomal protein genes (Neely and Workman, 2002). Our lyticase sensitivity assay results revealed that the evolved strain had significantly higher cell wall integrity than the reference strain (Figure 10), which may be associated with the missense mutation in *RSC30*.

Thirteen of the 14 genes that were up-regulated in H7 by at least sevenfold were found to be regulated by *SUA7*, with nine of them regulated by *SPT10*. *SUA7* encodes a yeast transcription factor TFIIB homolog and it has a function in transcription start site selection (Pinto et al., 1992). In addition, *SPT10* is known as a global regulator that binds to the histone upstream activating sequence elements (Eriksson et al., 2005).

*AIM19, FIS1, IRC15, IKS1, GDH2, YGL052W, VBA1, FYV6, TPS1, VHS3*, and *HSP82* are genes which have a missense point mutation, and which are also up-regulated in H7. According to the regulation enrichment analysis results using YEASTRACT database (Teixeira et al., 2018), it was found that the transcription factors Yap1p, Gcn4p, and Met31p have regulatory control on all these genes. Yap1p is a basic leucine zipper transcription factor required for oxidative stress tolerance and is activated by H_2_O_2_ (Cherry et al., 2012). Gcn4p is a key transcriptional activator of amino acid biosynthesis genes, and it plays a role in longevity and stress response (Mittal et al., 2017). Met31p is a zinc-finger DNA-binding transcription factor involved in the regulation of the methionine biosynthesis genes (Cherry et al., 2012). Among the genes regulated by these transcription factors, *IKS1* encodes a putative serine–threonine kinase and it is an Ira1p kinase suppressor. Deletion of *IKS1* gene causes hypersensitivity to copper sulfate (Dastidar et al., 2012) and a sorbate- resistant phenotype (Rieger et al., 1999). *IKS1* may be involved in the regulation of key pathways in acute stress defense (Ma et al., 2015). In H7, the mutation that occurred in the protein kinase domain of the protein encoded by *IKS1* could be important for oxidative stress resistance. Gdh2p, the NAD^+^-dependent GDH (NAD-GDH; Gdh2) encoded by *GDH2*, catalyzes reversible oxidative deamination of glutamate to α-ketoglutarate and ammonia (Miller and Magasanik, 1990). Gdh2-dependent NAD^+^ supply stimulates the growth at low temperature (Ballester-Tomás et al., 2015). In H7 genome, the mutation occurred in a region encoding the Leu/Phe/Val dehydrogenases active site of this gene. To our knowledge, there are no previous studies that describe such a mutation which warrants further investigation.

In this study, we successfully generated an oxidative stress-resistant and genetically stable *S. cerevisiae* strain *via* an evolutionary engineering strategy with heat pretreatment. Due to the nature of the stress and pretreatment condition, this strain is resistant to heat, freeze-thaw, ethanol, salt, iron, and cobalt stress conditions. Underlying mechanistic changes providing this adaptive resistance were elucidated with (i) differences in glycogen degradation, maltose, trehalose, glycerol, and acetate production levels; (ii) lyticase sensitivity assay that strongly supports the potential role of the cell wall in oxidative stress resistance of the evolved strain; (iii) comparative transcriptomic analysis showing changes in expression profiles in genes associated with autophagy, carbon metabolism, peroxisome and cell wall organization or biogenesis; (iv) comparative genomic analysis of the evolved strain showing mutations in diverse genes which are under the control of critical transcription factors. Investigation of the individual effects of the identified mutations in H7 genome *via* genome editing and reverse engineering strategies will be key toward a better understanding of the complex molecular mechanisms of oxidative stress resistance and specific genomic player(s). For this purpose, incorporation of the identified mutations in the reference strain as single mutations and/or their combinations, using novel and powerful genome editing strategies such as CRISPR-Cas9 is planned as the reverse engineering approach.

## Data Availability Statement

The datasets presented in this study can be found in online repositories. The names of the repository/repositories and accession number(s) can be found in the article/Supplementary Material.

## Author Contributions

ZÇ: conceptualization, supervision, and funding acquisition. NK-Ö, BY, CA, MA, AT, HK, and EG: methodology. NK-Ö and BY: resources. NK-Ö, BY, and CA: data curation and writing – original draft preparation. NK-Ö, BY, and ZÇ: writing and review and editing and project administration. All authors contributed to the article and approved the submitted version.

## Conflict of Interest

The authors declare that the research was conducted in the absence of any commercial or financial relationships that could be construed as a potential conflict of interest.

## Publisher’s Note

All claims expressed in this article are solely those of the authors and do not necessarily represent those of their affiliated organizations, or those of the publisher, the editors and the reviewers. Any product that may be evaluated in this article, or claim that may be made by its manufacturer, is not guaranteed or endorsed by the publisher.

## Footnotes

1
http://www.yeastgenome.org

2
https://www.ncbi.nlm.nih.gov/geo/query/acc.cgi?acc=GSE184952
