# Supplementary material for: Physiological and Molecular Characterization of an Oxidative Stress-Resistant Saccharomyces cerevisiae Strain Obtained by Evolutionary Engineering
Source: Front Microbiol. 2022 Feb 24;13:822864. doi: 10.3389/fmicb.2022.822864 (PMC8911705; doi:10.3389/fmicb.2022.822864)
Supplement: Supplementary file 1 [file Table_1.DOCX]

**Table S1.** Genes that were up-regulated in the evolved strain H7 by at least 2-fold, compared to the reference strain.

| **Process Name** | **Systematic Gene Symbol** | **Standard Gene Symbol** | **Gene Name** | **Fold Change** |
| --- | --- | --- | --- | --- |
| carbohydrate metabolic process | YGL156W | *AMS1* | Alpha-MannoSidase | 3.32 |
|  | YBR149W | *ARA1* | D-ARAbinose dehydrogenase | 2.34 |
|  | YPR026W | *ATH1* | Acid TreHalase | 3.25 |
|  | YMR280C | *CAT8* | CATabolite repression | 4.1 |
|  | YLR377C | *FBP1* | Fructose-1,6-BisPhosphatase | 3.1 |
|  | YIL097W | *FYV10* | Function required for Yeast Viability | 2.78 |
|  | YOR178C | *GAC1* | Glycogen ACcumulation | 3.78 |
|  | YLR081W | *GAL2* | GALactose metabolism | 3.33 |
|  | YBR018C | *GAL7* | GALactose metabolism | 4.22 |
|  | YOR120W | *GCY1* | Galactose-inducible Crystallin-like Yeast protein | 3.86 |
|  | YPR184W | *GDB1* | Glycogen DeBranching | 3.24 |
|  | YER054C | *GIP2* | Glc7-Interacting Protein | 3.67 |
|  | YEL011W | *GLC3* | GLyCogen | 4.28 |
|  | YCL040W | *GLK1* | GLucoKinase | 3.9 |
|  | YPR160W | *GPH1* | Glycogen PHosphorylase | 6.83 |
|  | YHR104W | *GRE3* | Genes de Respuesta a Estres (stress responsive genes) | 2.78 |
|  | YFR015C | *GSY1* | Glycogen SYnthase | 4.76 |
|  | YLR258W | *GSY2* | Glycogen SYnthase | 3.55 |
|  | YIL155C | *GUT2* | Glycerol UTilization | 4.07 |
|  | YFR053C | *HXK1* | HeXoKinase | 7.63 |
|  | YER065C | *ICL1* | IsoCitrate Lyase | 2.72 |
|  | YFR017C | *IGD1* | Inhibitor of Glycogen Debranching | 4.55 |
|  | YGR289C | *MAL11* | MALtose fermentation | 3.94 |
|  | YGR292W | *MAL12* | MALtose fermentation | 4.85 |
|  | YBR299W | *MAL32* | MALtose | 4.51 |
|  | YOL126C | *MDH2* | Malate DeHydrogenase | 2.82 |
|  | YNL117W | *MLS1* | MaLate Synthase | 3.16 |
|  | YDL085W | *NDE2* | NADH Dehydrogenase, External | 4.16 |
|  | YDR001C | *NTH1* | Neutral TreHalase | 2.37 |
|  | YOL032W | *OPI10* | OverProducer of Inositol | 2.61 |
|  | YKR097W | *PCK1* | Phosphoenolpyruvate CarboxyKinase | 3.55 |
|  | YMR105C | *PGM2* | PhosphoGlucoMutase | 5.33 |
|  | YDR255C | *RMD5* | Required for Meiotic nuclear Division | 2.56 |
|  | YDR511W | *SDH7* | Succinate DeHydrogenase | 2.36 |
|  | YIL099W | *SGA1* | Sporulation-specific GlycoAmylase | 4.84 |
|  | YJL089W | *SIP4* | SNF1-Interacting Protein | 2.53 |
|  | YBR126C | *TPS1* | Trehalose-6-Phosphate Synthase | 2.45 |
|  | YDR074W | *TPS2* | Trehalose-6-Phosphate Synthase/phosphatase | 2.98 |
|  | YML100W | *TSL1* | Trehalose Synthase Long chain | 5.39 |
|  | YEL012W | *UBC8* | UBiquitin-Conjugating | 2.99 |
|  | YKL035W | *UGP1* | UDP-glucose pyrophosphorylase | 2.63 |
|  | YGR194C | *XKS1* | XyluloKinaSe | 3.0 |
|  | YLR070C | *XYL2* |  | 2.44 |
|  | YJR096W | *YJR096W* |  | 4.27 |
|  | YLR345W | *YLR345W* |  | 2.61 |
| response to chemical | YDR216W | *ADR1* | Alcohol Dehydrogenase II synthesis Regulator | 3.08 |
|  | YPR026W | *ATH1* | Acid TreHalase | 3.25 |
|  | YNL305C | *BXI1* | BaX Inhibitor | 2.62 |
|  | YMR280C | *CAT8* | CATabolite repression | 4.1 |
|  | YKR066C | *CCP1* | Cytochrome c Peroxidase | 2.48 |
|  | YOR028C | *CIN5* | Chromosome INstability | 4.51 |
|  | YGR088W | *CTT1* | CaTalase T | 5.37 |
|  | YHR053C | *CUP1-1* | Cu, copper, CUPrum | 2.77 |
|  | YHR055C | *CUP1-2* | Cu, copper, CUPrum | 2.7 |
|  | YOL052C-A | *DDR2* | DNA Damage Responsive | 7.0 |
|  | YMR250W | *GAD1* | GlutAmate Decarboxylase | 4.03 |
|  | YOR120W | *GCY1* | Galactose-inducible Crystallin-like Yeast protein | 3.86 |
|  | YKL026C | *GPX1* | Glutathione PeroXidase | 3.45 |
|  | YHR104W | *GRE3* | Genes de Respuesta a Estres (stress responsive genes) | 2.78 |
|  | YCL035C | *GRX1* | GlutaRedoXin | 3.91 |
|  | YDR513W | *GRX2* | GlutaRedoXin | 3.59 |
|  | YDL223C | *HBT1* | HuB1 Target | 3.99 |
|  | YFL014W | *HSP12* | Heat Shock Protein | 8.44 |
|  | YCR091W | *KIN82* | protein KINase | 2.51 |
|  | YKL150W | *MCR1* | Mitochondrial NADH-Cytochrome b5 Reductase | 2.5 |
|  | YNL036W | *NCE103* | NonClassical Export | 4.86 |
|  | YGR043C | *NQM1* | Non-Quiescent Mutant | 6.36 |
|  | YBR066C | *NRG2* | Negative Regulator of Glucose-controlled genes | 2.52 |
|  | YDR001C | *NTH1* | Neutral TreHalase | 2.37 |
|  | YPL196W | *OXR1* | OXidation Resistance | 2.39 |
|  | YDR406W | *PDR15* | Pleiotropic Drug Resistance | 2.7 |
|  | YDL214C | *PRR2* | Pheromone Response Regulator | 5.77 |
|  | YBL064C | *PRX1* | PeroxiRedoXin | 3.73 |
|  | YOL117W | *RRI2* |  | 2.68 |
|  | YMR175W | *SIP18* | Salt Induced Protein | 4.56 |
|  | YJL089W | *SIP4* | SNF1-Interacting Protein | 2.53 |
|  | YKL086W | *SRX1* | SulfiRedoXin | 4.31 |
|  | YGR008C | *STF2* | STabilizing Factor | 3.36 |
|  | YBR126C | *TPS1* | Trehalose-6-Phosphate Synthase | 2.45 |
|  | YDR453C | *TSA2* | Thiol-Specific Antioxidant | 3.45 |
|  | YPL230W | *USV1* | Up in StarVation | 4.53 |
|  | YIL101C | *XBP1* | XhoI site-Binding Protein | 4.12 |
|  | YJL144W | *YJL144W* |  | 3.82 |
|  | YJR096W | *YJR096W* |  | 4.27 |
|  | YBR046C | *ZTA1* | ZeTA-crystallin | 3.11 |
| generation of precursor metabolites and energy | YNR001C | *CIT1* | CITrate synthase | 2.34 |
|  | Q0105 | *COB* | CytochrOme B | 2.56 |
|  | Q0045 | *COX1* | Cytochrome c OXidase | 2.65 |
|  | YIL111W | *COX5B* | Cytochrome c OXidase | 3.99 |
|  | YMR256C | *COX7* | Cytochrome c OXidase | 2.82 |
|  | YEL039C | *CYC7* | CYtochrome C | 9.09 |
|  | YBR026C | *ETR1* | 2-Enoyl Thioester Reductase | 3.31 |
|  | YOR178C | *GAC1* | Glycogen ACcumulation | 3.78 |
|  | YPR184W | *GDB1* | Glycogen DeBranching | 3.24 |
|  | YER054C | *GIP2* | Glc7-Interacting Protein | 3.67 |
|  | YEL011W | *GLC3* | GLyCogen | 4.28 |
|  | YCL040W | *GLK1* | GLucoKinase | 3.9 |
|  | YPR160W | *GPH1* | Glycogen PHosphorylase | 6.83 |
|  | YFR015C | *GSY1* | Glycogen SYnthase | 4.76 |
|  | YLR258W | *GSY2* | Glycogen SYnthase | 3.55 |
|  | YFR053C | *HXK1* | HeXoKinase | 7.63 |
|  | YFR017C | *IGD1* | Inhibitor of Glycogen Debranching | 4.55 |
|  | YMR081C | *ISF1* | Increasing Suppression Factor | 5.82 |
|  | YKL093W | *MBR1* | Mitochondrial Biogenesis Regulation | 5.41 |
|  | YDL085W | *NDE2* | NADH Dehydrogenase, External | 4.16 |
|  | YML120C | *NDI1* | NADH Dehydrogenase Internal | 2.37 |
|  | YMR105C | *PGM2* | PhosphoGlucoMutase | 5.33 |
|  | YHR001W-A | *QCR10* | ubiQuinol-cytochrome C oxidoReductase | 2.55 |
|  | YGR183C | *QCR9* | ubiQuinol-cytochrome C oxidoReductase | 2.37 |
|  | YER067W | *RGI1* | Respiratory Growth Induced | 6.0 |
|  | YIL057C | *RGI2* | Respiratory growth induced | 7.69 |
|  | YIL099W | *SGA1* | Sporulation-specific GlycoAmylase | 4.84 |
|  | YLR164W | *SHH4* | SDH4 Homolog | 2.86 |
|  | YKL035W | *UGP1* | UDP-glucose pyrophosphorylase | 2.63 |
|  | YLR345W | *YLR345W* |  | 2.61 |
|  | YFR049W | *YMR31* | Yeast Mitochondrial Ribosomal protein | 2.54 |
| nucleobase-containing small molecule metabolic process | YOR374W | *ALD4* | ALdehyde Dehydrogenase | 5.22 |
|  | Q0105 | *COB* | CytochrOme B | 2.56 |
|  | Q0045 | *COX1* | Cytochrome c OXidase | 2.65 |
|  | YIL111W | *COX5B* | Cytochrome c OXidase | 3.99 |
|  | YMR256C | *COX7* | Cytochrome c OXidase | 2.82 |
|  | YEL039C | *CYC7* | CYtochrome C | 9.09 |
|  | YCL040W | *GLK1* | GLucoKinase | 3.9 |
|  | YGR256W | *GND2* | 6-phosphoGlucoNateDehydrogenase | 4.73 |
|  | YDL022W | *GPD1* | Glycerol-3-Phosphate Dehydrogenase | 2.56 |
|  | YIL155C | *GUT2* | Glycerol UTilization | 4.07 |
|  | YFR053C | *HXK1* | HeXoKinase | 7.63 |
|  | YDL085W | *NDE2* | NADH Dehydrogenase, External | 4.16 |
|  | YML120C | *NDI1* | NADH Dehydrogenase Internal | 2.37 |
|  | YER037W | *PHM8* | PHosphate Metabolism | 2.58 |
|  | YGL037C | *PNC1* | Pyrazinamidase and NiCotinamidase | 2.96 |
|  | YHR001W-A | *QCR10* | ubiQuinol-cytochrome C oxidoReductase | 2.55 |
|  | YGR183C | *QCR9* | ubiQuinol-cytochrome C oxidoReductase | 2.37 |
|  | YLR164W | *SHH4* | SDH4 Homolog | 2.86 |
|  | YGR248W | *SOL4* | Suppressor Of Los1-1 | 5.17 |
|  | YBR117C | *TKL2* | TransKetoLase | 6.52 |
|  | YMR271C | *URA10* | URAcil requiring | 3.77 |
|  | YKL151C | *YKL151C* |  | 3.75 |
|  | YLR345W | *YLR345W* |  | 2.61 |
|  | YNL200C | *YNL200C* |  | 3.49 |
| response to oxidative stress | YKR066C | *CCP1* | Cytochrome c Peroxidase | 2.48 |
|  | YGR088W | *CTT1* | CaTalase T | 5.37 |
|  | YHR053C | *CUP1-1* | Cu, copper, CUPrum | 2.77 |
|  | YHR055C | *CUP1-2* | Cu, copper, CUPrum | 2.7 |
|  | YOL052C-A | *DDR2* | DNA Damage Responsive | 7.0 |
|  | YMR250W | *GAD1* | GlutAmate Decarboxylase | 4.03 |
|  | YOR120W | *GCY1* | Galactose-inducible Crystallin-like Yeast protein | 3.86 |
|  | YKL026C | *GPX1* | Glutathione PeroXidase | 3.45 |
|  | YHR104W | *GRE3* | Genes de Respuesta a Estres (stress responsive genes) | 2.78 |
|  | YCL035C | *GRX1* | GlutaRedoXin | 3.91 |
|  | YDR513W | *GRX2* | GlutaRedoXin | 3.59 |
|  | YFL014W | *HSP12* | Heat Shock Protein | 8.44 |
|  | YKL150W | *MCR1* | Mitochondrial NADH-Cytochrome b5 Reductase | 2.5 |
|  | YNL036W | *NCE103* | NonClassical Export | 4.86 |
|  | YGR043C | *NQM1* | Non-Quiescent Mutant | 6.36 |
|  | YPL196W | *OXR1* | OXidation Resistance | 2.39 |
|  | YBL064C | *PRX1* | PeroxiRedoXin | 3.73 |
|  | YKL086W | *SRX1* | SulfiRedoXin | 4.31 |
|  | YBR126C | *TPS1* | Trehalose-6-Phosphate Synthase | 2.45 |
|  | YDR453C | *TSA2* | Thiol-Specific Antioxidant | 3.45 |
|  | YIL101C | *XBP1* | XhoI site-Binding Protein | 4.12 |
|  | YJR096W | *YJR096W* |  | 4.27 |
|  | YBR046C | *ZTA1* | ZeTA-crystallin | 3.11 |
| transmembrane transport | YCR010C | *ADY2* | Accumulation of DYads | 6.24 |
|  | YBR132C | *AGP2* | high-Affinity Glutamine Permease | 3.31 |
|  | YPR192W | *AQY1* | AQuaporin from Yeast | 3.89 |
|  | YDR039C | *ENA2* | Exitus NAtru (Latin, "exit sodium") | 5.22 |
|  | YDL022W | *GPD1* | Glycerol-3-Phosphate Dehydrogenase | 2.56 |
|  | YDL245C | *HXT15* | HeXose Transporter | 2.8 |
|  | YDR343C | *HXT6* | HeXose Transporter | 6.38 |
|  | YDR342C | *HXT7* | HeXose Transporter | 6.3 |
|  | YKL217W | *JEN1* |  | 6.12 |
|  | YOL126C | *MDH2* | Malate DeHydrogenase | 2.82 |
|  | YGL080W | *MPC1* | Mitochondrial Pyruvate Carrier | 2.5 |
|  | YGR243W | *MPC3* | Mitochondrial Pyruvate Carrier | 6.77 |
|  | YNL214W | *PEX17* | PEroXisome related | 2.36 |
|  | YHR160C | *PEX18* | PEroXin | 4.02 |
|  | YAL005C | *SSA1* | Stress-Seventy subfamily A | 2.7 |
|  | YER103W | *SSA4* | Stress-Seventy subfamily A | 4.18 |
|  | YDR536W | *STL1* | Sugar Transporter-Like protein | 6.91 |
|  | YGL104C | *VPS73* | Vacuolar Protein Sorting | 2.58 |
|  | YFL054C | *AQY3* | AQuaporin from Yeast | 3.37 |
| meiotic cell cycle | YPR192W | *AQY1* | AQuaporin from Yeast | 3.89 |
|  | YPL200W | *CSM4* | Chromosome Segregation in Meiosis | 4.44 |
|  | YKL096W | *CWP1* | Cell Wall Protein | 4.64 |
|  | YDR516C | *EMI2* | Early Meiotic Induction | 4.81 |
|  | YDL222C | *FMP45* | Found in Mitochondrial Proteome | 7.89 |
|  | YOR178C | *GAC1* | Glycogen ACcumulation | 3.78 |
|  | YGL192W | *IME4* | Inducer of MEiosis | 3.19 |
|  | YPL017C | *IRC15* | Increased Recombination Centers | 4.28 |
|  | YOR351C | *MEK1* | MEiotic Kinase | 3.37 |
|  | YML128C | *MSC1* | Meiotic Sister-Chromatid recombination | 5.12 |
|  | YLR054C | *OSW2* | Outer Spore Wall | 3.05 |
|  | YHL024W | *RIM4* | Regulator of IME2 | 3.17 |
|  | YGL250W | *RMR1* | Reduced Meiotic Recombination | 2.98 |
|  | YOL048C | *RRT8* | Regulator of rDNA Transcription | 4.15 |
|  | YNL196C | *SLZ1* |  | 7.27 |
|  | YGR059W | *SPR3* | SPorulation Regulated | 2.6 |
|  | YLL039C | *UBI4* | Ubiquitin | 3.04 |
|  | YNL194C | *YNL194C* |  | 8.3 |
| monocarboxylic acid metabolic process | YBL015W | *ACH1* | Acetyl CoA Hydrolase | 2.72 |
|  | YDR216W | *ADR1* | Alcohol Dehydrogenase II synthesis Regulator | 3.08 |
|  | YOR374W | *ALD4* | ALdehyde Dehydrogenase | 5.22 |
|  | YML042W | *CAT2* | Carnitine AcetylTransferase | 3.52 |
|  | YMR280C | *CAT8* | CATabolite repression | 4.1 |
|  | YOR100C | *CRC1* | CaRnitine Carrier | 4.24 |
|  | YML054C | *CYB2* | CYtochrome B | 4.46 |
|  | YBR026C | *ETR1* | 2-Enoyl Thioester Reductase | 3.31 |
|  | YKR009C | *FOX2* | Fatty acid OXidation | 2.53 |
|  | YCL040W | *GLK1* | GLucoKinase | 3.9 |
|  | YML004C | *GLO1* | GLyOxalase | 3.38 |
|  | YFR053C | *HXK1* | HeXoKinase | 7.63 |
|  | YER065C | *ICL1* | IsoCitrate Lyase | 2.72 |
|  | YNL117W | *MLS1* | MaLate Synthase | 3.16 |
|  | YDL085W | *NDE2* | NADH Dehydrogenase, External | 4.16 |
|  | YIL160C | *POT1* | Peroxisomal Oxoacyl Thiolase | 5.23 |
|  | YLR345W | *YLR345W* |  | 2.61 |
| ion transport | YCR010C | *ADY2* | Accumulation of DYads | 6.24 |
|  | YBR132C | *AGP2* | high-Affinity Glutamine Permease | 3.31 |
|  | YNR002C | *ATO2* | Ammonia (Ammonium) Transport Outward | 5.99 |
|  | YGR142W | *BTN2* | BaTteN disease | 3.39 |
|  | YDR039C | *ENA2* | Exitus NAtru (Latin, "exit sodium") | 5.22 |
|  | YKL187C | *FAT3* | FATty acid transporter 3 | 2.42 |
|  | YKL217W | *JEN1* |  | 6.12 |
|  | YCR091W | *KIN82* | protein KINase | 2.51 |
|  | YPL060W | *MFM1* | Mrs2 Function Modulating factor | 2.33 |
|  | YGL080W | *MPC1* | Mitochondrial Pyruvate Carrier | 2.5 |
|  | YGR243W | *MPC3* | Mitochondrial Pyruvate Carrier | 6.77 |
|  | YKR052C | *MRS4* | Mitochondrial RNA Splicing | 2.36 |
|  | YOR348C | *PUT4* | Proline UTilization | 4.04 |
|  | YOR049C | *RSB1* | Resistance to Sphingoid long-chain Base | 3.79 |
|  | YJR095W | *SFC1* | Succinate-Fumarate Carrier | 5.05 |
|  | YLL055W | *YCT1* | Yeast Cysteine Transporter | 2.63 |
| cofactor metabolic process | YOR374W | *ALD4* | ALdehyde Dehydrogenase | 5.22 |
|  | YNR001C | *CIT1* | CITrate synthase | 2.34 |
|  | YCL040W | *GLK1* | GLucoKinase | 3.9 |
|  | YGR256W | *GND2* | 6-phosphoGlucoNateDehydrogenase | 4.73 |
|  | YDL022W | *GPD1* | Glycerol-3-Phosphate Dehydrogenase | 2.56 |
|  | YIL155C | *GUT2* | Glycerol UTilization | 4.07 |
|  | YFR053C | *HXK1* | HeXoKinase | 7.63 |
|  | YDL085W | *NDE2* | NADH Dehydrogenase, External | 4.16 |
|  | YML120C | *NDI1* | NADH Dehydrogenase Internal | 2.37 |
|  | YGL037C | *PNC1* | Pyrazinamidase and NiCotinamidase | 2.96 |
|  | YGR248W | *SOL4* | Suppressor Of Los1-1 | 5.17 |
|  | YBR117C | *TKL2* | TransKetoLase | 6.52 |
|  | YKL151C | *YKL151C* |  | 3.75 |
|  | YLR345W | *YLR345W* |  | 2.61 |
|  | YNL200C | *YNL200C* |  | 3.49 |
| protein targeting | YBR128C | *ATG14* | AuTophaGy related | 2.79 |
|  | YBL078C | *ATG8* | AuTophaGy related | 2.99 |
|  | YDR358W | *GGA1* | Golgi-localized, Gamma-adaptin ear homology, Arf-binding protein | 2.67 |
|  | YDL022W | *GPD1* | Glycerol-3-Phosphate Dehydrogenase | 2.56 |
|  | YPL240C | *HSP82* | Heat Shock Protein | 2.87 |
|  | YOL126C | *MDH2* | Malate DeHydrogenase | 2.82 |
|  | YBR230C | *OM14* | Outer Membrane Protein of 14 kDa | 5.18 |
|  | YNL214W | *PEX17* | PEroXisome related | 2.36 |
|  | YHR160C | *PEX18* | PEroXin | 4.02 |
|  | YHR136C | *SPL2* | Suppressor of PLc1 deletion | 3.32 |
|  | YAL005C | *SSA1* | Stress-Seventy subfamily A | 2.7 |
|  | YER103W | *SSA4* | Stress-Seventy subfamily A | 4.18 |
|  | YGL104C | *VPS73* | Vacuolar Protein Sorting | 2.58 |
|  | YNL093W | *YPT53* | Yeast Protein Two | 5.4 |
| cellular respiration | YNR001C | *CIT1* | CITrate synthase | 2.34 |
|  | Q0105 | *COB* | CytochrOme B | 2.56 |
|  | Q0045 | *COX1* | Cytochrome c OXidase | 2.65 |
|  | YIL111W | *COX5B* | Cytochrome c OXidase | 3.99 |
|  | YMR256C | *COX7* | Cytochrome c OXidase | 2.82 |
|  | YEL039C | *CYC7* | CYtochrome C | 9.09 |
|  | YBR026C | *ETR1* | 2-Enoyl Thioester Reductase | 3.31 |
|  | YMR081C | *ISF1* | Increasing Suppression Factor | 5.82 |
|  | YKL093W | *MBR1* | Mitochondrial Biogenesis Regulation | 5.41 |
|  | YML120C | *NDI1* | NADH Dehydrogenase Internal | 2.37 |
|  | YHR001W-A | *QCR10* | ubiQuinol-cytochrome C oxidoReductase | 2.55 |
|  | YGR183C | *QCR9* | ubiQuinol-cytochrome C oxidoReductase | 2.37 |
|  | YLR164W | *SHH4* | SDH4 Homolog | 2.86 |
|  | YFR049W | *YMR31* | Yeast Mitochondrial Ribosomal protein | 2.54 |
| transcription from RNA polymerase II promoter | YDR216W | *ADR1* | Alcohol Dehydrogenase II synthesis Regulator | 3.08 |
|  | YMR280C | *CAT8* | CATabolite repression | 4.1 |
|  | YOR028C | *CIN5* | Chromosome INstability | 4.51 |
|  | YPR030W | *CSR2* | Chs5 Spa2 Rescue | 4.55 |
|  | YDR516C | *EMI2* | Early Meiotic Induction | 4.81 |
|  | YDR096W | *GIS1* | GIg1-2 Suppressor | 2.36 |
|  | YIR017C | *MET28* | METhionine | 2.4 |
|  | YDR253C | *MET32* | METhionine requiring | 2.94 |
|  | YBR066C | *NRG2* | Negative Regulator of Glucose-controlled genes | 2.52 |
|  | YDL214C | *PRR2* | Pheromone Response Regulator | 5.77 |
|  | YBR050C | *REG2* | REsistance to Glucose repression | 3.89 |
|  | YJL089W | *SIP4* | SNF1-Interacting Protein | 2.53 |
|  | YPL230W | *USV1* | Up in StarVation | 4.53 |
|  | YIL101C | *XBP1* | XhoI site-Binding Protein | 4.12 |
| cellular amino acid metabolic process | YFL030W | *AGX1* | Alanine:Glyoxylate aminotrans(X)ferase | 3.87 |
|  | YMR169C | *ALD3* | ALdehyde Dehydrogenase | 6.75 |
|  | YHR137W | *ARO9* | AROmatic amino acid requiring | 3.74 |
|  | YPL111W | *CAR1* | Catabolism of ARginine | 3.53 |
|  | YLR438W | *CAR2* | Catabolism of ARginine | 3.2 |
|  | YNR001C | *CIT1* | CITrate synthase | 2.34 |
|  | YMR250W | *GAD1* | GlutAmate Decarboxylase | 4.03 |
|  | YAL062W | *GDH3* | Glutamate DeHydrogenase | 3.06 |
|  | YIR017C | *MET28* | METhionine | 2.4 |
|  | YDR253C | *MET32* | METhionine requiring | 2.94 |
|  | YGR087C | *PDC6* | Pyruvate DeCarboxylase | 2.71 |
|  | YLR142W | *PUT1* | Proline UTilization | 3.16 |
| mitochondrion organization | YHL021C | *AIM17* | Altered Inheritance rate of Mitochondria | 4.44 |
|  | YPL166W | *ATG29* | AuTophaGy related | 2.34 |
|  | YBL078C | *ATG8* | AuTophaGy related | 2.99 |
|  | YDR258C | *HSP78* | Heat Shock Protein | 3.26 |
|  | YPL240C | *HSP82* | Heat Shock Protein | 2.87 |
|  | YBR230C | *OM14* | Outer Membrane Protein of 14 kDa | 5.18 |
|  | YGR183C | *QCR9* | ubiQuinol-cytochrome C oxidoReductase | 2.37 |
|  | YDR379C-A | *SDH6* |  | 4.36 |
|  | YDR511W | *SDH7* | Succinate DeHydrogenase | 2.36 |
|  | YAL005C | *SSA1* | Stress-Seventy subfamily A | 2.7 |
|  | YFR049W | *YMR31* | Yeast Mitochondrial Ribosomal protein | 2.54 |
|  | YOR019W | *YOR019W* |  | 3.41 |
| lipid metabolic process | YDR216W | *ADR1* | Alcohol Dehydrogenase II synthesis Regulator | 3.08 |
|  | YCR068W | *ATG15* | AuTophaGy related | 2.92 |
|  | YGR110W | *CLD1* | CardioLipin-specific Deacylase | 3.36 |
|  | YOR100C | *CRC1* | CaRnitine Carrier | 4.24 |
|  | YBR026C | *ETR1* | 2-Enoyl Thioester Reductase | 3.31 |
|  | YKR009C | *FOX2* | Fatty acid OXidation | 2.53 |
|  | YDR096W | *GIS1* | GIg1-2 Suppressor | 2.36 |
|  | YKR067W | *GPT2* | Glycerol-3-Phosphate acylTransferase | 2.47 |
|  | YKL150W | *MCR1* | Mitochondrial NADH-Cytochrome b5 Reductase | 2.5 |
|  | YIL160C | *POT1* | Peroxisomal Oxoacyl Thiolase | 5.23 |
|  | YDR018C | *YDR018C* |  | 4.37 |
| oligosaccharide metabolic process | YGL156W | *AMS1* | Alpha-MannoSidase | 3.32 |
|  | YPR026W | *ATH1* | Acid TreHalase | 3.25 |
|  | YGR289C | *MAL11* | MALtose fermentation | 3.94 |
|  | YGR292W | *MAL12* | MALtose fermentation | 4.85 |
|  | YBR299W | *MAL32* | MALtose | 4.51 |
|  | YDR001C | *NTH1* | Neutral TreHalase | 2.37 |
|  | YMR105C | *PGM2* | PhosphoGlucoMutase | 5.33 |
|  | YBR126C | *TPS1* | Trehalose-6-Phosphate Synthase | 2.45 |
|  | YDR074W | *TPS2* | Trehalose-6-Phosphate Synthase/phosphatase | 2.98 |
|  | YML100W | *TSL1* | Trehalose Synthase Long chain | 5.39 |
|  | YKL035W | *UGP1* | UDP-glucose pyrophosphorylase | 2.63 |
| carbohydrate transport | YLR081W | *GAL2* | GALactose metabolism | 3.33 |
|  | YCL040W | *GLK1* | GLucoKinase | 3.9 |
|  | YFR053C | *HXK1* | HeXoKinase | 7.63 |
|  | YDL245C | *HXT15* | HeXose Transporter | 2.8 |
|  | YMR011W | *HXT2* | HeXose Transporter | 4.82 |
|  | YHR096C | *HXT5* | HeXose Transporter | 6.62 |
|  | YDR343C | *HXT6* | HeXose Transporter | 6.38 |
|  | YDR342C | *HXT7* | HeXose Transporter | 6.3 |
|  | YGR289C | *MAL11* | MALtose fermentation | 3.94 |
|  | YDR277C | *MTH1* | MSN Three Homolog | 4.22 |
|  | YDR536W | *STL1* | Sugar Transporter-Like protein | 6.91 |
| response to heat | YPR158W | *CUR1* | Curing of [URe3] | 2.66 |
|  | YOL052C-A | *DDR2* | DNA Damage Responsive | 7.0 |
|  | YOR178C | *GAC1* | Glycogen ACcumulation | 3.78 |
|  | YFL014W | *HSP12* | Heat Shock Protein | 8.44 |
|  | YBR072W | *HSP26* | Heat Shock Protein | 7.32 |
|  | YDR258C | *HSP78* | Heat Shock Protein | 3.26 |
|  | YPL004C | *LSP1* | Long chain bases Stimulate Phosphorylation | 2.41 |
|  | YDL079C | *MRK1* | Mds1p Related Kinase | 6.31 |
|  | YER103W | *SSA4* | Stress-Seventy subfamily A | 4.18 |
|  | YBR126C | *TPS1* | Trehalose-6-Phosphate Synthase | 2.45 |
|  | YDR074W | *TPS2* | Trehalose-6-Phosphate Synthase/phosphatase | 2.98 |
| protein phosphorylation | YJL057C | *IKS1* |  | 3.44 |
|  | YCR091W | *KIN82* | protein KINase | 2.51 |
|  | YPL004C | *LSP1* | Long chain bases Stimulate Phosphorylation | 2.41 |
|  | YOR351C | *MEK1* | MEiotic Kinase | 3.37 |
|  | YDL079C | *MRK1* | Mds1p Related Kinase | 6.31 |
|  | YDL214C | *PRR2* | Pheromone Response Regulator | 5.77 |
|  | YIL113W | *SDP1* | Stress-inducible Dual specificity Phosphatase | 3.42 |
|  | YGL208W | *SIP2* | SNF1-Interacting Protein | 2.36 |
|  | YMR291W | *TDA1* | Topoisomerase I Damage Affected | 2.4 |
|  | YJL164C | *TPK1* | Takashi's Protein Kinase | 2.94 |
| sporulation | YPR192W | *AQY1* | AQuaporin from Yeast | 3.89 |
|  | YKL096W | *CWP1* | Cell Wall Protein | 4.64 |
|  | YDR516C | *EMI2* | Early Meiotic Induction | 4.81 |
|  | YDL222C | *FMP45* | Found in Mitochondrial Proteome | 7.89 |
|  | YLR054C | *OSW2* | Outer Spore Wall | 3.05 |
|  | YHL024W | *RIM4* | Regulator of IME2 | 3.17 |
|  | YOL048C | *RRT8* | Regulator of rDNA Transcription | 4.15 |
|  | YGR059W | *SPR3* | SPorulation Regulated | 2.6 |
|  | YLL039C | *UBI4* | Ubiquitin | 3.04 |
|  | YNL194C |  |  | 8.3 |
| signaling | YNL305C | *BXI1* | BaX Inhibitor | 2.62 |
|  | YGL121C | *GPG1* | G Protein Gamma | 4.41 |
|  | YDR277C | *MTH1* | MSN Three Homolog | 4.22 |
|  | YGR070W | *ROM1* | RhO1 Multicopy suppressor | 2.48 |
|  | YOL117W | *RRI2* |  | 2.68 |
|  | YIL113W | *SDP1* | Stress-inducible Dual specificity Phosphatase | 3.42 |
|  | YGL208W | *SIP2* | SNF1-Interacting Protein | 2.36 |
|  | YIL105C | *SLM1* | Synthetic Lethal with Mss4 | 2.82 |
|  | YLR178C | *TFS1* | cdc Twenty-Five Suppressor | 4.54 |
|  | YJL164C | *TPK1* | Takashi's Protein Kinase | 2.94 |
| protein complex biogenesis | YPL240C | *HSP82* | Heat Shock Protein | 2.87 |
|  | YPL017C | *IRC15* | Increased Recombination Centers | 4.28 |
|  | YGL087C | *MMS2* | Methyl MethaneSulfonate sensitivity | 2.49 |
|  | YPR154W | *PIN3* | Psi+ INducibility | 2.49 |
|  | YGR183C | *QCR9* | ubiQuinol-cytochrome C oxidoReductase | 2.37 |
|  | YDR379C-A | *SDH6* |  | 4.36 |
|  | YDR511W | *SDH7* | Succinate DeHydrogenase | 2.36 |
|  | YGL208W | *SIP2* | SNF1-Interacting Protein | 2.36 |
|  | YDL110C | *TMA17* | Translation Machinery Associated | 3.54 |
| response to osmotic stress | YOR028C | *CIN5* | Chromosome INstability | 4.51 |
|  | YGR088W | *CTT1* | CaTalase T | 5.37 |
|  | YDL022W | *GPD1* | Glycerol-3-Phosphate Dehydrogenase | 2.56 |
|  | YHR104W | *GRE3* | Genes de Respuesta a Estres (stress responsive genes) | 2.78 |
|  | YFL014W | *HSP12* | Heat Shock Protein | 8.44 |
|  | YPL240C | *HSP82* | Heat Shock Protein | 2.87 |
|  | YDL079C | *MRK1* | Mds1p Related Kinase | 6.31 |
|  | YBR066C | *NRG2* | Negative Regulator of Glucose-controlled genes | 2.52 |
|  | YPL230W | *USV1* | Up in StarVation | 4.53 |
| organelle fission | YPL200W | *CSM4* | Chromosome Segregation in Meiosis | 4.44 |
|  | YOR178C | *GAC1* | Glycogen ACcumulation | 3.78 |
|  | YGL192W | *IME4* | Inducer of MEiosis | 3.19 |
|  | YPL017C | *IRC15* | Increased Recombination Centers | 4.28 |
|  | YOR351C | *MEK1* | MEiotic Kinase | 3.37 |
|  | YML128C | *MSC1* | Meiotic Sister-Chromatid recombination | 5.12 |
|  | YHL024W | *RIM4* | Regulator of IME2 | 3.17 |
|  | YGL250W | *RMR1* | Reduced Meiotic Recombination | 2.98 |
|  | YNL196C | *SLZ1* |  | 7.27 |
| protein modification by small protein conjugation or removal | YNL077W | *APJ1* | Anti-Prion DnaJ | 3.05 |
|  | YOR178C | *GAC1* | Glycogen ACcumulation | 3.78 |
|  | YGL087C | *MMS2* | Methyl MethaneSulfonate sensitivity | 2.49 |
|  | YDR255C | *RMD5* | Required for Meiotic nuclear Division | 2.56 |
|  | YOL117W | *RRI2* |  | 2.68 |
|  | YAL005C | *SSA1* | Stress-Seventy subfamily A | 2.7 |
|  | YEL012W | *UBC8* | UBiquitin-Conjugating | 2.99 |
|  | YLL039C | *UBI4* | Ubiquitin | 3.04 |
|  | YPL003W | *ULA1* | Ubiquitin-Like protein Activation | 2.44 |
| cell wall organization or biogenesis | YPR030W | *CSR2* | Chs5 Spa2 Rescue | 4.55 |
|  | YKL096W | *CWP1* | Cell Wall Protein | 4.64 |
|  | YDL222C | *FMP45* | Found in Mitochondrial Proteome | 7.89 |
|  | YLR054C | *OSW2* | Outer Spore Wall | 3.05 |
|  | YOL048C | *RRT8* | Regulator of rDNA Transcription | 4.15 |
|  | YIL113W | *SDP1* | Stress-inducible Dual specificity Phosphatase | 3.42 |
|  | YNL160W | *YGP1* | Yeast GlycoProtein | 4.52 |
|  | YIR039C | *YPS6* | YaPSin | 3.82 |
| peroxisome organization | YDR216W | *ADR1* | Alcohol Dehydrogenase II synthesis Regulator | 3.08 |
|  | YBR128C | *ATG14* | AuTophaGy related | 2.79 |
|  | YJL185C | *ATG36* | AuTophaGy related | 3.36 |
|  | YDL022W | *GPD1* | Glycerol-3-Phosphate Dehydrogenase | 2.56 |
|  | YKL026C | *GPX1* | Glutathione PeroXidase | 3.45 |
|  | YOL126C | *MDH2* | Malate DeHydrogenase | 2.82 |
|  | YNL214W | *PEX17* | PEroXisome related | 2.36 |
|  | YHR160C | *PEX18* | PEroXin | 4.02 |
| regulation of organelle organization | YDR216W | *ADR1* | Alcohol Dehydrogenase II synthesis Regulator | 3.08 |
|  | YPL200W | *CSM4* | Chromosome Segregation in Meiosis | 4.44 |
|  | YOR178C | *GAC1* | Glycogen ACcumulation | 3.78 |
|  | YPL240C | *HSP82* | Heat Shock Protein | 2.87 |
|  | YOR351C | *MEK1* | MEiotic Kinase | 3.37 |
|  | YPR154W | *PIN3* | Psi+ INducibility | 2.49 |
|  | YIL101C | *XBP1* | XhoI site-Binding Protein | 4.12 |
| protein folding | YGR142W | *BTN2* | BaTteN disease | 3.39 |
|  | YPR158W | *CUR1* | Curing of [URe3] | 2.66 |
|  | YBR072W | *HSP26* | Heat Shock Protein | 7.32 |
|  | YDR258C | *HSP78* | Heat Shock Protein | 3.26 |
|  | YPL240C | *HSP82* | Heat Shock Protein | 2.87 |
|  | YAL005C | *SSA1* | Stress-Seventy subfamily A | 2.7 |
|  | YER103W | *SSA4* | Stress-Seventy subfamily A | 4.18 |
| DNA recombination | YPL200W | *CSM4* | Chromosome Segregation in Meiosis | 4.44 |
|  | YPL017C | *IRC15* | Increased Recombination Centers | 4.28 |
|  | YML128C | *MSC1* | Meiotic Sister-Chromatid recombination | 5.12 |
|  | YDL059C | *RAD59* | RADiation sensitive | 2.43 |
|  | YHL024W | *RIM4* | Regulator of IME2 | 3.17 |
|  | YGL250W | *RMR1* | Reduced Meiotic Recombination | 2.98 |
| cytoskeleton organization | YDR171W | *HSP42* | Heat Shock Protein | 2.83 |
|  | YPL017C | *IRC15* | Increased Recombination Centers | 4.28 |
|  | YPR154W | *PIN3* | Psi+ INducibility | 2.49 |
|  | YIL105C | *SLM1* | Synthetic Lethal with Mss4 | 2.82 |
|  | YHR016C | *YSC84* |  | 2.74 |
| regulation of cell cycle | YPL200W | *CSM4* | Chromosome Segregation in Meiosis | 4.44 |
|  | YOR178C | *GAC1* | Glycogen ACcumulation | 3.78 |
|  | YPL017C | *IRC15* | Increased Recombination Centers | 4.28 |
|  | YOR351C | *MEK1* | MEiotic Kinase | 3.37 |
|  | YDR253C | *MET32* | METhionine requiring | 2.94 |
| proteolysis involved in cellular protein catabolic process | YIL097W | *FYV10* | Function required for Yeast Viability | 2.78 |
|  | YOR178C | *GAC1* | Glycogen ACcumulation | 3.78 |
|  | YDR358W | *GGA1* | Golgi-localized, Gamma-adaptin ear homology, Arf-binding protein | 2.67 |
|  | YDR255C | *RMD5* | Required for Meiotic nuclear Division | 2.56 |
|  | YEL012W | *UBC8* | UBiquitin-Conjugating | 2.99 |
| Golgi vesicle transport | YBL078C | *ATG8* | AuTophaGy related | 2.99 |
|  | YLR080W | *EMP46* |  | 3.23 |
|  | YDR358W | *GGA1* | Golgi-localized, Gamma-adaptin ear homology, Arf-binding protein | 2.67 |
|  | YGR209C | *TRX2* | ThioRedoXin | 2.79 |
|  | YNL093W | *YPT53* | Yeast Protein Two | 5.4 |
| invasive growth in response to glucose limitation | YDL024C | *DIA3* | Digs Into Agar | 4.05 |
|  | YGL121C | *GPG1* | G Protein Gamma | 4.41 |
|  | YBR066C | *NRG2* | Negative Regulator of Glucose-controlled genes | 2.52 |
|  | YGL208W | *SIP2* | SNF1-Interacting Protein | 2.36 |
|  | YJL089W | *SIP4* | SNF1-Interacting Protein | 2.53 |
| endocytosis | YPR030W | *CSR2* | Chs5 Spa2 Rescue | 4.55 |
|  | YPL004C | *LSP1* | Long chain bases Stimulate Phosphorylation | 2.41 |
|  | YBR214W | *SDS24* | homolog of S. pombe SDS23 | 3.64 |
|  | YNL093W | *YPT53* | Yeast Protein Two | 5.4 |
|  | YHR016C | *YSC84* |  | 2.74 |
| cellular response to DNA damage stimulus | YOL052C-A | *DDR2* | DNA Damage Responsive | 7.0 |
|  | YMR173W | *DDR48* | DNA Damage Responsive | 2.5 |
|  | YGL087C | *MMS2* | Methyl MethaneSulfonate sensitivity | 2.49 |
|  | YDL059C | *RAD59* | RADiation sensitive | 2.43 |
| conjugation | YDL223C | *HBT1* | HuB1 Target | 3.99 |
|  | YCR091W | *KIN82* | protein KINase | 2.51 |
|  | YDL214C | *PRR2* | Pheromone Response Regulator | 5.77 |
|  | YOL117W | *RRI2* |  | 2.68 |
| regulation of protein modification process | YOR178C | *GAC1* | Glycogen ACcumulation | 3.78 |
|  | YPL004C | *LSP1* | Long chain bases Stimulate Phosphorylation | 2.41 |
|  | YIL113W | *SDP1* | Stress-inducible Dual specificity Phosphatase | 2.68 |
|  | YIL101C | *XBP1* | XhoI site-Binding Protein | 4.12 |
| chromatin organization | YDR216W | *ADR1* | Alcohol Dehydrogenase II synthesis Regulator | 3.08 |
|  | YDR096W | *GIS1* | GIg1-2 Suppressor | 2.36 |
|  | YGL037C | *PNC1* | Pyrazinamidase and NiCotinamidase | 2.96 |
|  | YIL101C | *XBP1* | XhoI site-Binding Protein | 4.12 |
| endosomal transport | YGR142W | *BTN2* | BaTteN disease | 3.39 |
|  | YDR358W | *GGA1* | Golgi-localized, Gamma-adaptin ear homology, Arf-binding protein | 2.67 |
|  | YIL105C | *SLM1* | Synthetic Lethal with Mss4 | 2.82 |
| membrane invagination | YBL078C | *ATG8* | AuTophaGy related | 2.99 |
|  | YBR128C | *ATG14* | AuTophaGy related | 2.79 |
|  | YCR068W | *ATG15* | AuTophaGy related | 2.92 |
| response to starvation | YDR096W | *GIS1* | GIg1-2 Suppressor | 2.36 |
|  | YER037W | *PHM8* | PHosphate Metabolism | 2.58 |
|  | YGL208W | *SIP2* | SNF1-Interacting Protein | 2.36 |
| vacuole organization | YBL078C | *ATG8* | AuTophaGy related | 2.99 |
|  | YGR209C | *TRX2* | ThioRedoXin | 2.79 |
|  | YHR138C | *YHR138C* |  | 3.63 |
| mitotic cell cycle | YOR178C | *GAC1* | Glycogen ACcumulation | 3.78 |
|  | YDR253C | *MET32* | METhionine requiring | 2.94 |
|  | YGL229C | *SAP4* | Sit4 Associated Protein | 2.88 |
| pseudohyphal growth | YDL024C | *DIA3* | Digs Into Agar | 4.05 |
|  | YGL192W | *IME4* | Inducer of MEiosis | 3.19 |
|  | YBR066C | *NRG2* | Negative Regulator of Glucose-controlled genes | 2.52 |
| membrane fusion | YBL078C | *ATG8* | AuTophaGy related | 2.99 |
|  | YGR209C | *TRX2* | ThioRedoXin | 2.79 |
|  | YHR138C | *YHR138C* |  | 3.63 |
| lipid transport | YKL187C | *FAT3* | FATty acid transporter 3 | 2.42 |
|  | YCR091W | *KIN82* | protein KINase | 2.51 |
|  | YOR049C | *RSB1* | Resistance to Sphingoid long-chain Base | 3.79 |
| DNA repair | YMR173W | *DDR48* | DNA Damage Responsive | 2.5 |
|  | YGL087C | *MMS2* | Methyl MethaneSulfonate sensitivity | 2.49 |
|  | YDL059C | *RAD59* | RADiation sensitive | 2.43 |
| RNA catabolic process | YLR270W | *DCS1* | DeCapping Scavenger | 2.55 |
|  | YOR173W | *DCS2* | DeCapping Scavenger | 4.76 |
|  | YPL123C | *RNY1* | RiboNuclease from Yeast | 3.3 |
| chromosome segregation | YPL200W | *CSM4* | Chromosome Segregation in Meiosis | 4.44 |
|  | YOR178C | *GAC1* | Glycogen ACcumulation | 3.78 |
|  | YPL017C | *IRC15* | Increased Recombination Centers | 4.28 |
| regulation of DNA metabolic process | YPL200W | *CSM4* | Chromosome Segregation in Meiosis | 4.44 |
|  | YPL240C | *HSP82* | Heat Shock Protein | 2.87 |
|  | YGL037C | *PNC1* | Pyrazinamidase and NiCotinamidase | 2.96 |
| amino acid transport | YGR142W | *BTN2* | BaTteN disease | 3.39 |
|  | YOR348C | *PUT4* | Proline UTilization | 4.04 |
|  | YLL055W | *YCT1* | Yeast Cysteine Transporter | 2.63 |
| organelle fusion | YNL015W | *PBI2* | Proteinase B Inhibitor | 3.63 |
|  | YGR209C | *TRX2* | ThioRedoXin | 2.79 |
|  | YHR138C | *YHR138C* |  | 3.63 |
| RNA modification | YGL192W | *IME4* | Inducer of MEiosis | 3.19 |
|  | YNL196C | *SLZ1* |  | 7.27 |
| histone modification | YDR096W | *GIS1* | GIg1-2 Suppressor | 2.36 |
|  | YIL101C | *XBP1* | XhoI site-Binding Protein | 4.12 |
| cell morphogenesis | YDL223C | *HBT1* | HuB1 Target | 3.99 |
|  | YPL123C | *RNY1* | RiboNuclease from Yeast | 3.3 |
| DNA replication | YPL240C | *HSP82* | Heat Shock Protein | 2.87 |
|  | YHL024W | *RIM4* | Regulator of IME2 | 3.17 |
| nuclear transport | YNR034W | *SOL1* | Suppressor Of Los1-1 | 2.38 |
|  | YAL005C | *SSA1* | Stress-Seventy subfamily A | 2.7 |
| telomere organization | YDL059C | *RAD59* | RADiation sensitive | 2.43 |
|  | YPL240C | *HSP82* | Heat Shock Protein | 2.87 |
| nucleus organization | YOR185C | *GSP2* | Genetic Suppressor of Prp20-1 | 2.63 |
| organelle assembly | YBL078C | *ATG8* | AuTophaGy related | 2.99 |
| peptidyl-amino acid modification | YNL077W | *APJ1* | Anti-Prion DnaJ | 3.05 |
| organelle inheritance | YGR209C | *TRX2* | ThioRedoXin | 2.79 |
| cellular ion homeostasis | YMR105C | *PGM2* | PhosphoGlucoMutase | 5.33 |
| protein alkylation | YNL092W | *YNL092W* |  | 2.62 |
| nucleobase-containing compound transport | YNR034W | *SOL1* | Suppressor Of Los1-1 | 2.38 |
| mitochondrial translation | YFR049W | *YMR31* | Yeast Mitochondrial Ribosomal protein | 2.54 |
| regulation of transport | YBR132C | *AGP2* | high-Affinity Glutamine Permease | 3.31 |
| protein dephosphorylation | YER054C | *GIP2* | Glc7-Interacting Protein | 3.67 |
| protein maturation | YCR068W | *ATG15* | AuTophaGy related | 2.92 |
| cytoplasmic translation | YAL005C | *SSA1* | Stress-Seventy subfamily A | 2.7 |
