# Supplementary material for: Physiological and Molecular Characterization of an Oxidative Stress-Resistant Saccharomyces cerevisiae Strain Obtained by Evolutionary Engineering
Source: Front Microbiol. 2022 Feb 24;13:822864. doi: 10.3389/fmicb.2022.822864 (PMC8911705; doi:10.3389/fmicb.2022.822864)
Supplement: Supplementary file 2 [file Table_2.DOCX]

**Table S2.** Genes that were down-regulated in the evolved strain H7 by at least 2-fold, compared to the reference strain.

| **Process Name** | **Systematic Gene Symbol** | **Standard Gene Symbol** | **Gene Name** | **Fold Change** |
| --- | --- | --- | --- | --- |
| rRNA processing | YDR299W | *BFR2* | BreFeldin A Resistance | 3.31 |
|  | YPL217C | *BMS1* | BMh Sensitive | 3.31 |
|  | YIL096C | *BMT5* | Base Methyltransferase of Twenty five S rRNA 5 | 3.05 |
|  | YMR014W | *BUD22* | BUD site selection | 2.49 |
|  | YCR047C | *BUD23* | BUD site selection | 2.39 |
|  | YLR175W | *CBF5* | Centromere Binding Factor | 3.61 |
|  | YGL029W | *CGR1* | Coiled-coil Growth-Regulated | 2.72 |
|  | YDL031W | *DBP10* | Dead Box Protein | 2.57 |
|  | YNL112W | *DBP2* | Dead Box Protein | 5.24 |
|  | YGL078C | *DBP3* | Dead Box Protein | 3.18 |
|  | YKR024C | *DBP7* | Dead Box Protein | 2.93 |
|  | YHR169W | *DBP8* | Dead Box Protein | 3.48 |
|  | YLR276C | *DBP9* | Dead Box Protein | 3.14 |
|  | YKL078W | *DHR2* | DEAH-box RNA helicase | 4.33 |
|  | YPL266W | *DIM1* | DIMethylase | 2.54 |
|  | YLR129W | *DIP2* | DOM34 Interacting Protein | 2.58 |
|  | YLL008W | *DRS1* | Deficiency of Ribosomal Subunits | 3.46 |
|  | YKL172W | *EBP2* | EBNA1-binding protein (homolog) | 2.75 |
|  | YMR128W | *ECM16* | ExtraCellular Mutant | 2.88 |
|  | YGR271C-A | *EFG1* | Exit From G1 | 2.66 |
|  | YLR186W | *EMG1* | Essential for Mitotic Growth | 2.54 |
|  | YBR247C | *ENP1* | Essential Nuclear Protein | 2.81 |
|  | YGR145W | *ENP2* | Essential Nuclear Protein | 3.03 |
|  | YMR049C | *ERB1* | Eukaryotic Ribosome Biogenesis | 3.02 |
|  | YNR054C | *ESF2* | Eighteen S rRNA Factor 2 | 2.8 |
|  | YIL019W | *FAF1* | Forty (40) S Assembly Factor | 2.62 |
|  | YDR021W | *FAL1* | eukaryotic translation initiation factor Four A Like | 2.59 |
|  | YLR068W | *FYV7* | Function required for Yeast Viability | 2.71 |
|  | YHR089C | *GAR1* | Glycine Arginine Rich | 3.1 |
|  | YLL035W | *GRC3* |  | 4.53 |
|  | YMR290C | *HAS1* | Helicase Associated with Set1 | 3.29 |
|  | YJL033W | *HCA4* | Helicase CA | 3.25 |
|  | YHR148W | *IMP3* | Interacting with Mpp10p | 2.45 |
|  | YNL075W | *IMP4* | Interacting with Mpp10p | 3.14 |
|  | YHR085W | *IPI1* | Involved in Processing ITS2 | 2.85 |
|  | YNL182C | *IPI3* | Involved in Processing ITS2 | 3.57 |
|  | YCL059C | *KRR1* | contains KRR-R motif | 2.62 |
|  | YER127W | *LCP5* | Lethal with Conditional Pap1 | 2.84 |
|  | YAL025C | *MAK16* | MAintenance of Killer | 3.37 |
|  | YBR142W | *MAK5* | MAintenance of Killer | 2.81 |
|  | YJR002W | *MPP10* | M Phase Phosphoproteins | 3.0 |
|  | YPR112C | *MRD1* | Multiple RNA-binding domain | 2.71 |
|  | YKL009W | *MRT4* | mRNA Turnover 4 | 3.06 |
|  | YJL050W | *MTR4* | Mrna TRansport | 2.83 |
|  | YPL211W | *NIP7* | Nuclear ImPort | 3.02 |
|  | YLR002C | *NOC3* | NucleOlar Complex associated | 2.63 |
|  | YPR144C | *NOC4* | NucleOlar Complex associated | 2.44 |
|  | YPL093W | *NOG1* | NucleOlar G-protein | 3.58 |
|  | YDL014W | *NOP1* | NucleOlar Protein | 3.33 |
|  | YOL041C | *NOP12* | NucleOlar Protein | 2.86 |
|  | YNL061W | *NOP2* | NucleOlar Protein | 3.73 |
|  | YPL043W | *NOP4* | NucleOlar Protein | 3.14 |
|  | YLR197W | *NOP56* | NucleOlar Protein of 56.8 kDa | 2.41 |
|  | YOR310C | *NOP58* | NucleOlar Protein of 58 kDa | 3.55 |
|  | YGR103W | *NOP7* | NucleOlar Protein | 3.13 |
|  | YOL144W | *NOP8* | NucleOlar Protein | 2.66 |
|  | YJL010C | *NOP9* | NucleOlar Protein | 3.24 |
|  | YER126C | *NSA2* | Nop Seven Associated | 2.54 |
|  | YGR159C | *NSR1* |  | 4.81 |
|  | YER006W | *NUG1* | NUclear GTPase | 2.98 |
|  | YOR145C | *PNO1* | Partner of NOb1 | 2.55 |
|  | YGL120C | *PRP43* | Pre-mRNA Processing | 2.78 |
|  | YOR243C | *PUS7* | PseudoUridine Synthase | 2.55 |
|  | YLR196W | *PWP1* | Periodic tryptophan (W) Protein | 2.85 |
|  | YCR057C | *PWP2* | Periodic tryptophan (W) Protein | 3.06 |
|  | YOL010W | *RCL1* | Rna 3'-terminal phosphate Cyclase Like | 2.99 |
|  | YOL080C | *REX4* | Rna EXonuclease | 2.85 |
|  | YHR197W | *RIX1* | RIbosome eXport | 2.71 |
|  | YGL171W | *ROK1* | Rescuer Of Kem1 | 2.67 |
|  | YKR081C | *RPF2* | Ribosome Production Factor | 2.85 |
|  | YHR062C | *RPP1* | Ribonuclease P Protein | 2.4 |
|  | YPL012W | *RRP12* | Ribosomal RNA Processing | 3.18 |
|  | YOR287C | *RRP36* | Ribosomal RNA Processing | 2.44 |
|  | YMR229C | *RRP5* | Ribosomal RNA Processing | 2.93 |
|  | YDR083W | *RRP8* | Ribosomal RNA Processing | 3.12 |
|  | YPR137W | *RRP9* | Ribosomal RNA Processing | 2.72 |
|  | YOR294W | *RRS1* | Regulator of Ribosome Synthesis | 3.59 |
|  | YDL153C | *SAS10* | Something About Silencing | 2.45 |
|  | YEL026W | *SNU13* | Small NUclear ribonucleoprotein associated | 3.64 |
|  | YLL011W | *SOF1* | Suppressor Of Fibrillarin | 2.42 |
|  | YCL054W | *SPB1* | Suppressor of PaB1 mutant | 2.8 |
|  | YFL002C | *SPB4* | Suppressor of PAB1 | 2.43 |
|  | YNL209W | *SSB2* | Stress-Seventy subfamily B | 2.39 |
|  | YHR066W | *SSF1* | Suppressor of ste4 (Four) | 2.58 |
|  | YPR016C | *TIF6* | Translation Initiation Factor | 2.34 |
|  | YDL060W | *TSR1* | Twenty S rRNA accumulation | 2.7 |
|  | YLR435W | *TSR2* | Twenty S rRNA accumulation | 2.65 |
|  | YJL109C | *UTP10* | U Three Protein | 2.6 |
|  | YLR222C | *UTP13* | U Three Protein | 2.84 |
|  | YML093W | *UTP14* | U Three Protein | 2.4 |
|  | YMR093W | *UTP15* | U Three Protein | 2.39 |
|  | YJL069C | *UTP18* | U Three Protein | 2.74 |
|  | YLR409C | *UTP21* | U Three Protein | 2.76 |
|  | YOR004W | *UTP23* | U Three-associated Protein | 3.82 |
|  | YIL091C | *UTP25* | U Three Protein | 2.9 |
|  | YDR324C | *UTP4* | U Three Protein | 2.82 |
|  | YDR398W | *UTP5* | U Three Protein | 3.25 |
|  | YDR449C | *UTP6* | U Three Protein | 2.55 |
|  | YGR128C | *UTP8* | U Three Protein | 2.75 |
| ribosomal small subunit biogenesis | YDR299W | *BFR2* | BreFeldin A Resistance | 3.31 |
|  | YPL217C | *BMS1* | BMh Sensitive | 3.31 |
|  | YMR014W | *BUD22* | BUD site selection | 2.49 |
|  | YCR047C | *BUD23* | BUD site selection | 2.39 |
|  | YHR169W | *DBP8* | Dead Box Protein | 3.48 |
|  | YKL078W | *DHR2* | DEAH-box RNA helicase | 4.33 |
|  | YPL266W | *DIM1* | DIMethylase | 2.54 |
|  | YLR129W | *DIP2* | DOM34 Interacting Protein | 2.58 |
|  | YMR128W | *ECM16* | ExtraCellular Mutant | 2.88 |
|  | YGR271C-A | *EFG1* | Exit From G1 | 2.66 |
|  | YLR186W | *EMG1* | Essential for Mitotic Growth | 2.54 |
|  | YBR247C | *ENP1* | Essential Nuclear Protein | 2.81 |
|  | YGR145W | *ENP2* | Essential Nuclear Protein | 3.03 |
|  | YNR054C | *ESF2* | Eighteen S rRNA Factor 2 | 2.8 |
|  | YIL019W | *FAF1* | Forty (40) S Assembly Factor | 2.62 |
|  | YDR021W | *FAL1* | eukaryotic translation initiation factor Four A Like | 2.59 |
|  | YLR068W | *FYV7* | Function required for Yeast Viability | 2.71 |
|  | YMR290C | *HAS1* | Helicase Associated with Set1 | 3.29 |
|  | YHR148W | *IMP3* | Interacting with Mpp10p | 2.45 |
|  | YNL075W | *IMP4* | Interacting with Mpp10p | 3.14 |
|  | YNL132W | *KRE33* | Killer toxin REsistant | 4.37 |
|  | YCL059C | *KRR1* | contains KRR-R motif | 2.62 |
|  | YER127W | *LCP5* | Lethal with Conditional Pap1 | 2.84 |
|  | YJR002W | *MPP10* | M Phase Phosphoproteins | 3.0 |
|  | YPR112C | *MRD1* | Multiple RNA-binding domain | 2.71 |
|  | YPL226W | *NEW1* | Nu+ | 2.88 |
|  | YPR144C | *NOC4* | NucleOlar Complex associated | 2.44 |
|  | YOR310C | *NOP58* | NucleOlar Protein of 58 kDa | 3.55 |
|  | YGR103W | *NOP7* | NucleOlar Protein | 3.13 |
|  | YJL010C | *NOP9* | NucleOlar Protein | 3.24 |
|  | YGR159C | *NSR1* |  | 4.81 |
|  | YOR145C | *PNO1* | Partner of NOb1 | 2.55 |
|  | YGL120C | *PRP43* | Pre-mRNA Processing | 2.78 |
|  | YCR057C | *PWP2* | Periodic tryptophan (W) Protein | 3.06 |
|  | YOL010W | *RCL1* | Rna 3'-terminal phosphate Cyclase Like | 2.99 |
|  | YGL171W | *ROK1* | Rescuer Of Kem1 | 2.67 |
|  | YPL012W | *RRP12* | Ribosomal RNA Processing | 3.18 |
|  | YOR287C | *RRP36* | Ribosomal RNA Processing | 2.44 |
|  | YMR229C | *RRP5* | Ribosomal RNA Processing | 2.93 |
|  | YOR294W | *RRS1* | Regulator of Ribosome Synthesis | 3.59 |
|  | YDL153C | *SAS10* | Something About Silencing | 2.45 |
|  | YLR336C | *SGD1* | Suppressor of Glycerol Defect | 2.74 |
|  | YEL026W | *SNU13* | Small NUclear ribonucleoprotein associated | 3.64 |
|  | YLL011W | *SOF1* | Suppressor Of Fibrillarin | 2.42 |
|  | YDL060W | *TSR1* | Twenty S rRNA accumulation | 2.7 |
|  | YLR435W | *TSR2* | Twenty S rRNA accumulation | 2.65 |
|  | YJL109C | *UTP10* | U Three Protein | 2.6 |
|  | YLR222C | *UTP13* | U Three Protein | 2.84 |
|  | YML093W | *UTP14* | U Three Protein | 2.4 |
|  | YMR093W | *UTP15* | U Three Protein | 2.39 |
|  | YJL069C | *UTP18* | U Three Protein | 2.74 |
|  | YLR409C | *UTP21* | U Three Protein | 2.76 |
|  | YOR004W | *UTP23* | U Three-associated Protein | 3.82 |
|  | YIL091C | *UTP25* | U Three Protein | 2.9 |
|  | YDR324C | *UTP4* | U Three Protein | 2.82 |
|  | YDR398W | *UTP5* | U Three Protein | 3.25 |
|  | YDR449C | *UTP6* | U Three Protein | 2.55 |
|  | YGR128C | *UTP8* | U Three Protein | 2.75 |
| ribosomal large subunit biogenesis | YLR397C | *AFG2* | ATPase Family Gene | 2.87 |
|  | YJL122W | *ALB1* | Arx1 Little Brother | 4.04 |
|  | YOL077C | *BRX1* | Xenopus laevis Brix (Biogenesis of Ribosomes in Xenopus) homolog | 2.75 |
|  | YDL031W | *DBP10* | Dead Box Protein | 2.57 |
|  | YGL078C | *DBP3* | Dead Box Protein | 3.18 |
|  | YKR024C | *DBP7* | Dead Box Protein | 2.93 |
|  | YLR276C | *DBP9* | Dead Box Protein | 3.14 |
|  | YLL008W | *DRS1* | Deficiency of Ribosomal Subunits | 3.46 |
|  | YMR049C | *ERB1* | Eukaryotic Ribosome Biogenesis | 3.02 |
|  | YLL035W | *GRC3* |  | 4.53 |
|  | YMR290C | *HAS1* | Helicase Associated with Set1 | 3.29 |
|  | YHR085W | *IPI1* | Involved in Processing ITS2 | 2.85 |
|  | YNL182C | *IPI3* | Involved in Processing ITS2 | 3.57 |
|  | YGL099W | *LSG1* | Large-Subunit Gtpase | 2.51 |
|  | YAL025C | *MAK16* | MAintenance of Killer | 3.37 |
|  | YDR060W | *MAK21* | MAintenance of Killer | 3.14 |
|  | YBR142W | *MAK5* | MAintenance of Killer | 2.81 |
|  | YKL009W | *MRT4* | mRNA Turnover 4 | 3.06 |
|  | YPL211W | *NIP7* | Nuclear ImPort | 3.02 |
|  | YOR206W | *NOC2* | NucleOlar Complex associated | 2.5 |
|  | YPL093W | *NOG1* | NucleOlar G-protein | 3.58 |
|  | YOL041C | *NOP12* | NucleOlar Protein | 2.86 |
|  | YNL061W | *NOP2* | NucleOlar Protein | 3.73 |
|  | YPL043W | *NOP4* | NucleOlar Protein | 3.14 |
|  | YGR103W | *NOP7* | NucleOlar Protein | 3.13 |
|  | YOL144W | *NOP8* | NucleOlar Protein | 2.66 |
|  | YER126C | *NSA2* | Nop Seven Associated | 2.54 |
|  | YER006W | *NUG1* | NUclear GTPase | 2.98 |
|  | YGL120C | *PRP43* | Pre-mRNA Processing | 2.78 |
|  | YDR496C | *PUF6* | PUmilio-homology domain Family | 2.98 |
|  | YBR267W | *REI1* | REquired for Isotropic bud growth | 3.18 |
|  | YOL080C | *REX4* | Rna EXonuclease | 2.85 |
|  | YHR197W | *RIX1* | RIbosome eXport | 2.71 |
|  | YLL034C | *RIX7* | RIbosome eXport | 3.27 |
|  | YDR091C | *RLI1* | RNase L Inhibitor | 2.73 |
|  | YLR009W | *RLP24* | Ribosomal-Like Protein | 3.97 |
|  | YKR081C | *RPF2* | Ribosome Production Factor | 2.85 |
|  | YMR229C | *RRP5* | Ribosomal RNA Processing | 2.93 |
|  | YDR083W | *RRP8* | Ribosomal RNA Processing | 3.12 |
|  | YOR294W | *RRS1* | Regulator of Ribosome Synthesis | 3.59 |
|  | YCR072C | *RSA4* | RiboSome Assembly | 4.53 |
|  | YGR245C | *SDA1* | Severe Depolymerization of Actin | 5.7 |
|  | YCL054W | *SPB1* | Suppressor of PaB1 mutant | 2.8 |
|  | YFL002C | *SPB4* | Suppressor of PAB1 | 2.43 |
|  | YIR012W | *SQT1* | Suppressor of QSR1 Truncations | 2.43 |
|  | YHR066W | *SSF1* | Suppressor of ste4 (Four) | 2.58 |
|  | YDL063C | *SYO1* | SYnchronized impOrt or SYmpOrtin | 3.39 |
|  | YPR016C | *TIF6* | Translation Initiation Factor | 2.34 |
|  | YOR272W | *YTM1* |  | 3.44 |
|  | YIR026C | *YVH1* | Yeast vaccinia virus VH1 Homolog | 3.14 |
| RNA modification | YIL096C | *BMT5* | Base Methyltransferase of Twenty five S rRNA 5 | 3.05 |
|  | YCR047C | *BUD23* | BUD site selection | 2.39 |
|  | YLR175W | *CBF5* | Centromere Binding Factor | 3.61 |
|  | YPL266W | *DIM1* | DIMethylase | 2.54 |
|  | YML080W | *DUS1* | DihydroUridine Synthase | 2.37 |
|  | YGR200C | *ELP2* | ELongator Protein | 2.66 |
|  | YLR186W | *EMG1* | Essential for Mitotic Growth | 2.54 |
|  | YHR089C | *GAR1* | Glycine Arginine Rich | 3.1 |
|  | YNL062C | *GCD10* | General Control Derepressed | 2.81 |
|  | YLR384C | *IKI3* | Insensitive to KIller toxin | 2.46 |
|  | YBL024W | *NCL1* | NuCLear protein | 2.81 |
|  | YDL014W | *NOP1* | NucleOlar Protein | 3.33 |
|  | YNL061W | *NOP2* | NucleOlar Protein | 3.73 |
|  | YLR197W | *NOP56* | NucleOlar Protein of 56.8 kDa | 2.41 |
|  | YPL212C | *PUS1* | PseudoUridine Synthase | 2.78 |
|  | YOR243C | *PUS7* | PseudoUridine Synthase | 2.55 |
|  | YDR083W | *RRP8* | Ribosomal RNA Processing | 3.12 |
|  | YPR137W | *RRP9* | Ribosomal RNA Processing | 2.72 |
|  | YCL054W | *SPB1* | Suppressor of PaB1 mutant | 2.8 |
|  | YGL169W | *SUA5* | Suppressor of Upstream AUG | 2.36 |
|  | YDR120C | *TRM1* | tRNA Methyltransferase | 3.36 |
|  | YOL124C | *TRM11* | TRna Methyltransferase | 2.44 |
|  | YOL125W | *TRM13* | TRna Methyltransferase | 3.64 |
|  | YPL030W | *TRM44* | TRna Methyltransferase | 2.91 |
|  | YDL201W | *TRM8* | Transfer RNA Methyltransferase | 2.75 |
|  | YDR165W | *TRM82* | Transfer RNA Methyltransferase | 2.77 |
| nuclear transport | YDR101C | *ARX1* | Associated with Ribosomal eXport complex | 3.47 |
|  | YCR047C | *BUD23* | BUD site selection | 2.39 |
|  | YAL059W | *ECM1* | ExtraCellular Mutant | 2.41 |
|  | YBR034C | *HMT1* | HnRNP MethylTransferase | 3.8 |
|  | YER110C | *KAP123* | KAryoPherin | 3.35 |
|  | YGL099W | *LSG1* | Large-Subunit Gtpase | 2.51 |
|  | YPL226W | *NEW1* | Nu+ | 2.88 |
|  | YHR170W | *NMD3* | Nonsense-Mediated mRNA Decay | 2.36 |
|  | YPL093W | *NOG1* | NucleOlar G-protein | 3.58 |
|  | YNR053C | *NOG2* | NucleOlar G-protein | 3.82 |
|  | YJL010C | *NOP9* | NucleOlar Protein | 3.24 |
|  | YER006W | *NUG1* | NUclear GTPase | 2.98 |
|  | YBR267W | *REI1* | REquired for Isotropic bud growth | 3.18 |
|  | YHR197W | *RIX1* | RIbosome eXport | 2.71 |
|  | YLL034C | *RIX7* | RIbosome eXport | 3.27 |
|  | YDR091C | *RLI1* | RNase L Inhibitor | 2.73 |
|  | YOR294W | *RRS1* | Regulator of Ribosome Synthesis | 3.59 |
|  | YGR245C | *SDA1* | Severe Depolymerization of Actin | 5.7 |
|  | YKR092C | *SRP40* | Serine Rich Protein | 2.59 |
|  | YNL209W | *SSB2* | Stress-Seventy subfamily B | 2.39 |
|  | YDL063C | *SYO1* | SYnchronized impOrt or SYmpOrtin | 3.39 |
|  | YPR016C | *TIF6* | Translation Initiation Factor | 2.34 |
|  | YGR128C | *UTP8* | U Three Protein | 2.75 |
| organelle assembly | YOL077C | *BRX1* | Xenopus laevis Brix (Biogenesis of Ribosomes in Xenopus) homolog | 2.75 |
|  | YLL008W | *DRS1* | Deficiency of Ribosomal Subunits | 3.46 |
|  | YNR054C | *ESF2* | Eighteen S rRNA Factor 2 | 2.8 |
|  | YHR085W | *IPI1* | Involved in Processing ITS2 | 2.85 |
|  | YNL182C | *IPI3* | Involved in Processing ITS2 | 3.57 |
|  | YGL099W | *LSG1* | Large-Subunit Gtpase | 2.51 |
|  | YDR060W | *MAK21* | MAintenance of Killer | 3.14 |
|  | YPR112C | *MRD1* | Multiple RNA-binding domain | 2.71 |
|  | YKL009W | *MRT4* | mRNA Turnover 4 | 3.06 |
|  | YGR159C | *NSR1* |  | 4.81 |
|  | YOL080C | *REX4* | Rna EXonuclease | 2.85 |
|  | YHR197W | *RIX1* | RIbosome eXport | 2.71 |
|  | YKR081C | *RPF2* | Ribosome Production Factor | 2.85 |
|  | YCR072C | *RSA4* | RiboSome Assembly | 4.53 |
|  | YFL002C | *SPB4* | Suppressor of PAB1 | 2.43 |
|  | YIR012W | *SQT1* | Suppressor of QSR1 Truncations | 2.43 |
|  | YHR066W | *SSF1* | Suppressor of ste4 (Four) | 2.58 |
|  | YIR026C | *YVH1* | Yeast vaccinia virus VH1 Homolog | 3.14 |
| ribosome assembly | YOL077C | *BRX1* | Xenopus laevis Brix (Biogenesis of Ribosomes in Xenopus) homolog | 2.75 |
|  | YLL008W | *DRS1* | Deficiency of Ribosomal Subunits | 3.46 |
|  | YNR054C | *ESF2* | Eighteen S rRNA Factor 2 | 2.8 |
|  | YHR085W | *IPI1* | Involved in Processing ITS2 | 2.85 |
|  | YNL182C | *IPI3* | Involved in Processing ITS2 | 3.57 |
|  | YGL099W | *LSG1* | Large-Subunit Gtpase | 2.51 |
|  | YDR060W | *MAK21* | MAintenance of Killer | 3.14 |
|  | YPR112C | *MRD1* | Multiple RNA-binding domain | 2.71 |
|  | YKL009W | *MRT4* | mRNA Turnover 4 | 3.06 |
|  | YGR159C | *NSR1* |  | 4.81 |
|  | YOL080C | *REX4* | Rna EXonuclease | 2.85 |
|  | YHR197W | *RIX1* | RIbosome eXport | 2.71 |
|  | YKR081C | *RPF2* | Ribosome Production Factor | 2.85 |
|  | YCR072C | *RSA4* | RiboSome Assembly | 4.53 |
|  | YFL002C | *SPB4* | Suppressor of PAB1 | 2.43 |
|  | YIR012W | *SQT1* | Suppressor of QSR1 Truncations | 2.43 |
|  | YHR066W | *SSF1* | Suppressor of ste4 (Four) | 2.58 |
|  | YIR026C | *YVH1* | Yeast vaccinia virus VH1 Homolog | 3.14 |
| ribosomal subunit export from nucleus | YDR101C | *ARX1* | Associated with Ribosomal eXport complex | 3.47 |
|  | YCR047C | *BUD23* | BUD site selection | 2.39 |
|  | YAL059W | *ECM1* | ExtraCellular Mutant | 2.41 |
|  | YGL099W | *LSG1* | Large-Subunit Gtpase | 2.51 |
|  | YHR170W | *NMD3* | Nonsense-Mediated mRNA Decay | 2.36 |
|  | YPL093W | *NOG1* | NucleOlar G-protein | 3.58 |
|  | YNR053C | *NOG2* | NucleOlar G-protein | 3.82 |
|  | YJL010C | *NOP9* | NucleOlar Protein | 3.24 |
|  | YER006W | *NUG1* | NUclear GTPase | 2.98 |
|  | YHR197W | *RIX1* | RIbosome eXport | 2.71 |
|  | YLL034C | *RIX7* | RIbosome eXport | 3.27 |
|  | YDR091C | *RLI1* | RNase L Inhibitor | 2.73 |
|  | YOR294W | *RRS1* | Regulator of Ribosome Synthesis | 3.59 |
|  | YGR245C | *SDA1* | Severe Depolymerization of Actin | 5.7 |
|  | YNL209W | *SSB2* | Stress-Seventy subfamily B | 2.39 |
|  | YPR016C | *TIF6* | Translation Initiation Factor | 2.34 |
| tRNA processing | YML080W | *DUS1* | DihydroUridine Synthase | 2.37 |
|  | YGR200C | *ELP2* | ELongator Protein | 2.66 |
|  | YNL062C | *GCD10* | General Control Derepressed | 2.81 |
|  | YLR384C | *IKI3* | Insensitive to KIller toxin | 2.46 |
|  | YBL024W | *NCL1* | NuCLear protein | 2.81 |
|  | YPL212C | *PUS1* | PseudoUridine Synthase | 2.78 |
|  | YOR243C | *PUS7* | PseudoUridine Synthase | 2.55 |
|  | YHR062C | *RPP1* | Ribonuclease P Protein | 2.4 |
|  | YGL169W | *SUA5* | Suppressor of Upstream AUG | 2.36 |
|  | YDR120C | *TRM1* | tRNA Methyltransferase | 3.36 |
|  | YOL124C | *TRM11* | TRna Methyltransferase | 2.44 |
|  | YOL125W | *TRM13* | TRna Methyltransferase | 3.64 |
|  | YPL030W | *TRM44* | TRna Methyltransferase | 2.91 |
|  | YDL201W | *TRM8* | Transfer RNA Methyltransferase | 2.75 |
|  | YDR165W | *TRM82* | Transfer RNA Methyltransferase | 2.77 |
| transcription from RNA polymerase I promoter | YLL035W | *GRC3* |  | 4.53 |
|  | YPR010C | *RPA135* | RNA Polymerase A | 2.63 |
|  | YJL148W | *RPA34* | RNA Polymerase A | 2.32 |
|  | YOR340C | *RPA43* | RNA Polymerase A | 3.61 |
|  | YNL248C | *RPA49* | RNA Polymerase A | 2.85 |
|  | YBR154C | *RPB5* | RNA Polymerase B | 2.6 |
|  | YPR110C | *RPC40* | RNA Polymerase C | 2.52 |
|  | YJL109C | *UTP10* | U Three Protein | 2.6 |
|  | YMR093W | *UTP15* | U Three Protein | 2.39 |
|  | YDR324C | *UTP4* | U Three Protein | 2.82 |
|  | YDR398W | *UTP5* | U Three Protein | 3.25 |
|  | YGR128C | *UTP8* | U Three Protein | 2.75 |
| nucleobase-containing small molecule metabolic process | YNL141W | *AAH1* | Adenine AminoHydrolase | 4.13 |
|  | YMR300C | *ADE4* | ADEnine requiring | 2.44 |
|  | YGL234W | *ADE5,7* | ADEnine requiring | 2.94 |
|  | YMR217W | *GUA1* | GUanine Auxotroph | 2.6 |
|  | YLR432W | *IMD3* | IMP Dehydrogenase | 2.47 |
|  | YLR134W | *PDC5* | Pyruvate DeCarboxylase | 4.05 |
|  | YLR014C | *PPR1* | Pyrimidine Pathway Regulation | 2.45 |
|  | YOR101W | *RAS1* | homologous to RAS proto-oncogene | 3.02 |
|  | YOR095C | *RKI1* | Ribose-5-phosphate Ketol-Isomerase | 3.26 |
|  | YOR047C | *STD1* | Suppressor of Tbp Deletion | 2.65 |
|  | YKL216W | *URA1* | URAcil requiring | 2.73 |
|  | YBL039C | *URA7* | URAcil requiring | 3.36 |
| response to chemical | YDR161W | *ACL4* | Assembly Chaperone of RpL4 | 2.43 |
|  | YLR397C | *AFG2* | ATPase Family Gene | 2.87 |
|  | YGR177C | *ATF2* | AcetylTransFerase | 2.63 |
|  | YML116W | *ATR1* | AminoTriazole Resistance | 2.56 |
|  | YPL256C | *CLN2* | CycLiN | 2.51 |
|  | YGR271C-A | *EFG1* | Exit From G1 | 2.66 |
|  | YJL157C | *FAR1* | Factor ARrest | 2.59 |
|  | YMR052W | *FAR3* | Factor ARrest | 2.55 |
|  | YBL024W | *NCL1* | NuCLear protein | 2.81 |
|  | YDR091C | *RLI1* | RNase L Inhibitor | 2.73 |
|  | YFL026W | *STE2* | STErile | 2.5 |
| cellular amino acid metabolic process | YKL106W | *AAT1* | Aspartate AminoTransferase | 2.69 |
|  | YGL256W | *ADH4* | Alcohol DeHydrogenase | 5.36 |
|  | YDR321W | *ASP1* | ASParaginase | 2.64 |
|  | YNL256W | *FOL1* | FOLic acid synthesis | 2.37 |
|  | YER086W | *ILV1* | IsoLeucine-plus-Valine requiring | 2.55 |
|  | YJR016C | *ILV3* | IsoLeucine-plus-Valine requiring | 2.58 |
|  | YLR355C | *ILV5* | IsoLeucine-plus-Valine requiring | 2.42 |
|  | YOR108W | *LEU9* | LEUcine biosynthesis | 2.83 |
|  | YDR234W | *LYS4* | LYSine requiring | 2.94 |
|  | YLR134W | *PDC5* | Pyruvate DeCarboxylase | 4.05 |
|  | YHR020W | *YHR020W* |  | 2.56 |
| regulation of cell cycle | YBR158W | *AMN1* | Antagonist of Mitotic exit Network | 2.36 |
|  | YMR199W | *CLN1* | CycLiN | 2.83 |
|  | YPL256C | *CLN2* | CycLiN | 2.51 |
|  | YKR083C | *DAD2* | Duo1 And Dam1 interacting | 3.06 |
|  | YJL157C | *FAR1* | Factor ARrest | 2.59 |
|  | YDR130C | *FIN1* | Filaments In between Nuclei | 2.53 |
|  | YOR233W | *KIN4* | KINase | 3.21 |
|  | YNL289W | *PCL1* | Pho85 CycLin | 2.4 |
|  | YLR263W | *RED1* | REDuctional division | 2.46 |
|  | YGR245C | *SDA1* | Severe Depolymerization of Actin | 5.7 |
| mitotic cell cycle | YBR158W | *AMN1* | Antagonist of Mitotic exit Network | 2.36 |
|  | YDR184C | *ATC1* | Aip Three Complex | 2.79 |
|  | YCR047C | *BUD23* | BUD site selection | 2.39 |
|  | YCR063W | *BUD31* | BUD site selection | 2.39 |
|  | YDR130C | *FIN1* | Filaments In between Nuclei | 2.53 |
|  | YOR233W | *KIN4* | KINase | 3.21 |
|  | YBR267W | *REI1* | REquired for Isotropic bud growth | 3.18 |
|  | YDR180W | *SCC2* | Sister Chromatid Cohesion | 2.37 |
|  | YGR245C | *SDA1* | Severe Depolymerization of Actin | 5.7 |
|  | YOR315W | *SFG1* | SuperFicial pseudohyphal Growth | 2.73 |
| signaling | YPL256C | *CLN2* | CycLiN | 2.51 |
|  | YGR271C-A | *EFG1* | Exit From G1 | 2.66 |
|  | YJL157C | *FAR1* | Factor ARrest | 2.59 |
|  | YMR052W | *FAR3* | Factor ARrest | 2.55 |
|  | YDR144C | *MKC7* | Multicopy suppressor of Kex2 Cold sensitivity | 2.86 |
|  | YOR101W | *RAS1* | homologous to RAS proto-oncogene | 3.02 |
|  | YOR107W | *RGS2* | Regulator of heterotrimeric G protein Signaling | 2.82 |
|  | YOR047C | *STD1* | Suppressor of Tbp Deletion | 2.65 |
|  | YFL026W | *STE2* | STErile | 2.5 |
|  | YIR026C | *YVH1* | Yeast vaccinia virus VH1 Homolog | 3.14 |
| conjugation | YPL256C | *CLN2* | CycLiN | 2.51 |
|  | YGR271C-A | *EFG1* | Exit From G1 | 2.66 |
|  | YJL157C | *FAR1* | Factor ARrest | 2.59 |
|  | YMR052W | *FAR3* | Factor ARrest | 2.55 |
|  | YGL099W | *LSG1* | Large-Subunit Gtpase | 2.51 |
|  | YDL039C | *PRM7* | Pheromone-Regulated Membrane protein | 4.28 |
|  | YHR066W | *SSF1* | Suppressor of ste4 (Four) | 2.58 |
|  | YFL026W | *STE2* | STErile | 2.5 |
| RNA catabolic process | YIL079C | *AIR1* | Arginine methyltransferase-Interacting RING finger protein | 3.22 |
|  | YNL112W | *DBP2* | Dead Box Protein | 5.24 |
|  | YKL009W | *MRT4* | mRNA Turnover 4 | 3.06 |
|  | YJL050W | *MTR4* | Mrna TRansport | 2.83 |
|  | YJL208C | *NUC1* | NUClease | 2.56 |
|  | YHR062C | *RPP1* | Ribonuclease P Protein | 2.4 |
|  | YNL299W | *TRF5* | Topoisomerase one-Related Function | 2.59 |
|  | YOR359W | *VTS1* | VTi1-2 Suppressor | 2.35 |
| transcription from RNA polymerase II promoter | YER045C | *ACA1* | ATF/CREB Activator | 2.42 |
|  | YGR200C | *ELP2* | ELongator Protein | 2.66 |
|  | YBR034C | *HMT1* | HnRNP MethylTransferase | 3.8 |
|  | YLR384C | *IKI3* | Insensitive to KIller toxin | 2.46 |
|  | YLR014C | *PPR1* | Pyrimidine Pathway Regulation | 2.45 |
|  | YBR154C | *RPB5* | RNA Polymerase B | 2.6 |
|  | YOR047C | *STD1* | Suppressor of Tbp Deletion | 2.65 |
|  | YBL054W | *TOD6* | Twin Of Dot6p | 3.27 |
| organelle fission | YBR158W | *AMN1* | Antagonist of Mitotic exit Network | 2.36 |
|  | YKL172W | *EBP2* | EBNA1-binding protein (homolog) | 2.75 |
|  | YDR130C | *FIN1* | Filaments In between Nuclei | 2.53 |
|  | YOR233W | *KIN4* | KINase | 3.21 |
|  | YLR263W | *RED1* | REDuctional division | 2.46 |
|  | YDR180W | *SCC2* | Sister Chromatid Cohesion | 2.37 |
|  | YPL130W | *SPO19* | SPOrulation | 3.11 |
|  | YIR026C | *YVH1* | Yeast vaccinia virus VH1 Homolog | 3.14 |
| regulation of organelle organization | YBR158W | *AMN1* | Antagonist of Mitotic exit Network | 2.36 |
|  | YKR083C | *DAD2* | Duo1 And Dam1 interacting | 3.06 |
|  | YDR130C | *FIN1* | Filaments In between Nuclei | 2.53 |
|  | YLR449W | *FPR4* | FKBP Proline Rotamase (isomerase) | 3.01 |
|  | YOR233W | *KIN4* | KINase | 3.21 |
|  | YLR263W | *RED1* | REDuctional division | 2.46 |
|  | YIR026C | *YVH1* | Yeast vaccinia virus VH1 Homolog | 3.14 |
| transmembrane transport | YML116W | *ATR1* | AminoTriazole Resistance | 2.56 |
|  | YBR291C | *CTP1* | Citrate Transport Protein | 2.47 |
|  | YER056C | *FCY2* | FluoroCYtosine resistance | 3.16 |
|  | YBL042C | *FUI1* | 5-FlUorourIdine resistance | 3.56 |
|  | YJR054W | *KCH1* | Potassium (K) regulator of CcH1 | 2.9 |
|  | YBR104W | *YMC2* | Yeast Mitochondrial Carrier | 2.73 |
|  | YGL255W | *ZRT1* | Zinc-Regulated Transporter | 5.64 |
| peptidyl-amino acid modification | YHL039W | *EFM1* | Elongation Factor Methyltransferase | 3.42 |
|  | YBR271W | *EFM2* | Elongation Factor Methyltransferase | 3.72 |
|  | YIL064W | *EFM4* | Elongation Factor Methyltransferase | 2.58 |
|  | YLR449W | *FPR4* | FKBP Proline Rotamase (isomerase) | 3.01 |
|  | YBR034C | *HMT1* | HnRNP MethylTransferase | 3.8 |
|  | YJR070C | *LIA1* | Ligand of eIF5A | 2.71 |
|  | YDR465C | *RMT2* | aRginine MeThyltransferase | 3.02 |
| protein alkylation | YHL039W | *EFM1* | Elongation Factor Methyltransferase | 3.42 |
|  | YBR271W | *EFM2* | Elongation Factor Methyltransferase | 3.72 |
|  | YIL064W | *EFM4* | Elongation Factor Methyltransferase | 2.58 |
|  | YLR449W | *FPR4* | FKBP Proline Rotamase (isomerase) | 3.01 |
|  | YBR034C | *HMT1* | HnRNP MethylTransferase | 3.8 |
|  | YDL014W | *NOP1* | NucleOlar Protein | 3.33 |
|  | YDR465C | *RMT2* | aRginine MeThyltransferase | 3.02 |
| ion transport | YML116W | *ATR1* | AminoTriazole Resistance | 2.56 |
|  | YBR291C | *CTP1* | Citrate Transport Protein | 2.47 |
|  | YJR054W | *KCH1* | Potassium (K) regulator of CcH1 | 2.9 |
|  | YOR306C | *MCH5* | MonoCarboxylate transporter Homologue | 3.1 |
|  | YGR138C | *TPO2* | Transporter of POlyamines | 2.83 |
|  | YMR241W | *YHM2* | Yeast suppressor of HM mutant | 2.78 |
|  | YGL255W | *ZRT1* | Zinc-Regulated Transporter | 5.64 |
| DNA replication | YHR085W | *IPI1* | Involved in Processing ITS2 | 2.85 |
|  | YNL182C | *IPI3* | Involved in Processing ITS2 | 3.57 |
|  | YLR002C | *NOC3* | NucleOlar Complex associated | 2.63 |
|  | YGR103W | *NOP7* | NucleOlar Protein | 3.13 |
|  | YML060W | *OGG1* | 8-OxoGuanine Glycosylase/lyase | 2.61 |
|  | YHR197W | *RIX1* | RIbosome eXport | 2.71 |
| cytoskeleton organization | YKR083C | *DAD2* | Duo1 And Dam1 interacting | 3.06 |
|  | YDR130C | *FIN1* | Filaments In between Nuclei | 2.53 |
|  | YJR070C | *LIA1* | Ligand of eIF5A | 2.71 |
|  | YNL289W | *PCL1* | Pho85 CycLin | 2.4 |
|  | YGR245C | *SDA1* | Severe Depolymerization of Actin | 5.7 |
| mRNA processing | YCR063W | *BUD31* | BUD site selection | 2.39 |
|  | YMR268C | *PRP24* | Pre-mRNA Processing | 4.52 |
|  | YGL120C | *PRP43* | Pre-mRNA Processing | 2.78 |
|  | YMR061W | *RNA14* | poly(A) mRNA metabolism | 3.45 |
|  | YEL026W | *SNU13* | Small NUclear ribonucleoprotein associated | 3.64 |
| RNA splicing | Q0110 | *BI2* |  | 6.36 |
|  | YCR063W | *BUD31* | BUD site selection | 2.39 |
|  | YMR268C | *PRP24* | Pre-mRNA Processing | 4.52 |
|  | YGL120C | *PRP43* | Pre-mRNA Processing | 2.78 |
|  | YEL026W | *SNU13* | Small NUclear ribonucleoprotein associated | 3.64 |
| regulation of protein modification process | YMR199W | *CLN1* | CycLiN | 2.83 |
|  | YPL256C | *CLN2* | CycLiN | 2.51 |
|  | YDR130C | *FIN1* | Filaments In between Nuclei | 2.53 |
|  | YLR449W | *FPR4* | FKBP Proline Rotamase (isomerase) | 3.01 |
|  | YNL289W | *PCL1* | Pho85 CycLin | 2.4 |
| snoRNA processing | YLR175W | *CBF5* | Centromere Binding Factor | 3.61 |
|  | YJL050W | *MTR4* | Mrna TRansport | 2.83 |
|  | YDL014W | *NOP1* | NucleOlar Protein | 3.33 |
|  | YHR062C | *RPP1* | Ribonuclease P Protein | 2.4 |
|  | YNL299W | *TRF5* | Topoisomerase one-Related Function | 2.59 |
| generation of precursor metabolites and energy | YGL256W | *ADH4* | Alcohol DeHydrogenase | 5.36 |
|  | YGR177C | *ATF2* | AcetylTransFerase | 2.63 |
|  | YLR134W | *PDC5* | Pyruvate DeCarboxylase | 4.05 |
|  | YLR273C | *PIG1* | Protein Interacting with Gsy2p | 4.83 |
|  | YBR238C | *YBR238C* |  | 2.64 |
| chromosome segregation | YKR083C | *DAD2* | Duo1 And Dam1 interacting | 3.06 |
|  | YDR130C | *FIN1* | Filaments In between Nuclei | 2.53 |
|  | YOR233W | *KIN4* | KINase | 3.21 |
|  | YLR263W | *RED1* | REDuctional division | 2.46 |
|  | YDR180W | *SCC2* | Sister Chromatid Cohesion | 2.37 |
| nucleobase-containing compound transport | YER056C | *FCY2* | FluoroCYtosine resistance | 3.16 |
|  | YBL042C | *FUI1* | 5-FlUorourIdine resistance | 3.56 |
|  | YBR034C | *HMT1* | HnRNP MethylTransferase | 3.8 |
|  | YPL226W | *NEW1* | Nu+ | 2.88 |
|  | YGR128C | *UTP8* | U Three Protein | 2.75 |
| regulation of translation | YBR271W | *EFM2* | Elongation Factor Methyltransferase | 3.72 |
|  | YDR496C | *PUF6* | PUmilio-homology domain Family | 2.98 |
|  | YDR091C | *RLI1* | RNase L Inhibitor | 2.73 |
|  | YNL209W | *SSB2* | Stress-Seventy subfamily B | 2.39 |
|  | YGL169W | *SUA5* | Suppressor of Upstream AUG | 2.36 |
| protein phosphorylation | YMR199W | *CLN1* | CycLiN | 2.83 |
|  | YPL256C | *CLN2* | CycLiN | 2.51 |
|  | YOR233W | *KIN4* | KINase | 3.21 |
|  | YNL289W | *PCL1* | Pho85 CycLin | 2.4 |
| lipid metabolic process | YDR147W | *EKI1* | Ethanolamine KInase | 2.9 |
|  | YJL167W | *ERG20* | ERGosterol biosynthesis | 6.99 |
|  | YBL039C | *URA7* | URAcil requiring | 3.36 |
|  | YGR177C | *ATF2* | AcetylTransFerase | 2.63 |
| protein complex biogenesis | YFL026W | *STE2* | STErile | 2.5 |
|  | YOR145C | *PNO1* | Partner of NOb1 | 2.55 |
|  | YKR083C | *DAD2* | Duo1 And Dam1 interacting | 3.06 |
|  | YLR449W | *FPR4* | FKBP Proline Rotamase (isomerase) | 3.01 |
| meiotic cell cycle | YGL099W | *LSG1* | Large-Subunit Gtpase | 2.51 |
|  | YLR263W | *RED1* | REDuctional division | 2.46 |
|  | YPL130W | *SPO19* | SPOrulation | 3.11 |
|  | YIR026C | *YVH1* | Yeast vaccinia virus VH1 Homolog | 3.14 |
| cell wall organization or biogenesis | YNL313C | *EMW1* | Essential for Maintenance of the cell Wall | 2.55 |
|  | YDR144C | *MKC7* | Multicopy suppressor of Kex2 Cold sensitivity | 2.86 |
|  | YHL011C | *PRS3* | PhosphoRibosylpyrophosphate Synthetase | 2.52 |
|  | YIR026C | *YVH1* | Yeast vaccinia virus VH1 Homolog | 3.14 |
| regulation of DNA metabolic process | YHR085W | *IPI1* | Involved in Processing ITS2 | 2.85 |
|  | YNL182C | *IPI3* | Involved in Processing ITS2 | 3.57 |
|  | YHR197W | *RIX1* | RIbosome eXport | 2.71 |
|  | YOR359W | *VTS1* | VTi1-2 Suppressor | 2.35 |
| cytoplasmic translation | YAL036C | *RBG1* | RiBosome interacting Gtpase | 2.72 |
|  | YBR079C | *RPG1* |  | 3.43 |
|  | YCL037C | *SRO9* | Suppressor of rho3 | 2.42 |
|  | YNL209W | *SSB2* | Stress-Seventy subfamily B | 2.39 |
| cellular response to DNA damage stimulus | YER038C | *KRE29* | Killer toxin REsistant | 3.41 |
|  | YML060W | *OGG1* | 8-OxoGuanine Glycosylase/lyase | 2.61 |
|  | YDR180W | *SCC2* | Sister Chromatid Cohesion | 2.37 |
| carbohydrate metabolic process | YLR134W | *PDC5* | Pyruvate DeCarboxylase | 4.05 |
|  | YLR273C | *PIG1* | Protein Interacting with Gsy2p | 4.83 |
|  | YOR047C | *STD1* | Suppressor of Tbp Deletion | 2.65 |
| translational initiation | YDR091C | *RLI1* | RNase L Inhibitor | 2.73 |
|  | YBR079C | *RPG1* |  | 3.43 |
|  | YGR054W | *YGR054W* |  | 2.95 |
| DNA repair | YER038C | *KRE29* | Killer toxin REsistant | 3.41 |
|  | YML060W | *OGG1* | 8-OxoGuanine Glycosylase/lyase | 2.61 |
|  | YDR180W | *SCC2* | Sister Chromatid Cohesion | 2.37 |
| cytokinesis | YDR184C | *ATC1* | Aip Three Complex | 2.79 |
|  | YCR047C | *BUD23* | BUD site selection | 2.39 |
|  | YCR063W | *BUD31* | BUD site selection | 2.39 |
| transcription from RNA polymerase III promoter | YBR154C | *RPB5* | RNA Polymerase B | 2.6 |
|  | YPR110C | *RPC40* | RNA Polymerase C | 2.52 |
|  | YDL150W | *RPC53* | RNA Polymerase C | 3.93 |
| sporulation | YGL099W | *LSG1* | Large-Subunit Gtpase | 2.51 |
|  | YPL130W | *SPO19* | SPOrulation | 3.11 |
|  | YIR026C | *YVH1* | Yeast vaccinia virus VH1 Homolog | 3.14 |
| translational elongation | YNL209W | *SSB2* | Stress-Seventy subfamily B | 2.39 |
|  | YGL169W | *SUA5* | Suppressor of Upstream AUG | 2.36 |
|  | YLR249W | *YEF3* | Yeast Elongation Factor | 2.67 |
| proteolysis involved in cellular protein catabolic process | YDR161W | *ACL4* | Assembly Chaperone of RpL4 | 2.43 |
|  | YDR130C | *FIN1* | Filaments In between Nuclei | 2.53 |
|  | YOR233W | *KIN4* | KINase | 3.21 |
| cofactor metabolic process | YNL256W | *FOL1* | FOLic acid synthesis | 2.37 |
|  | YLR134W | *PDC5* | Pyruvate DeCarboxylase | 4.05 |
|  | YOR095C | *RKI1* | Ribose-5-phosphate Ketol-Isomerase | 3.26 |
| DNA-templated transcription, elongation | YBR034C | *HMT1* | HnRNP MethylTransferase | 3.8 |
|  | YJL148W | *RPA34* | RNA Polymerase A | 2.32 |
|  | YNL248C | *RPA49* | RNA Polymerase A | 2.85 |
| pseudohyphal growth | YLR083C | *EMP70* |  | 2.38 |
|  | YER110C | *KAP123* | KAryoPherin | 3.35 |
|  | YOR315W | *SFG1* | SuperFicial pseudohyphal Growth | 2.73 |
| DNA-templated transcription, termination | YLL035W | *GRC3* |  | 4.53 |
|  | YBR034C | *HMT1* | HnRNP MethylTransferase | 3.8 |
| protein targeting | YER110C | *KAP123* | KAryoPherin | 3.35 |
|  | YDL063C | *SYO1* | SYnchronized impOrt or SYmpOrtin | 3.39 |
| histone modification | YLR449W | *FPR4* | FKBP Proline Rotamase (isomerase) | 3.01 |
|  | YDL014W | *NOP1* | NucleOlar Protein | 3.33 |
| chromatin organization | YLR449W | *FPR4* | FKBP Proline Rotamase (isomerase) | 3.01 |
|  | YDL014W | *NOP1* | NucleOlar Protein | 3.33 |
| endosomal transport | YLR083C | *EMP70* |  | 2.38 |
|  | YPL183C | *RTT10* | Regulator of Ty1 Transposition | 2.83 |
| DNA recombination | YJL208C | *NUC1* | NUClease | 2.56 |
|  | YDR180W | *SCC2* | Sister Chromatid Cohesion | 2.37 |
| response to oxidative stress | YBL024W | *NCL1* | NuCLear protein | 2.81 |
|  | YDR091C | *RLI1* | RNase L Inhibitor | 2.73 |
| cell budding | YDR184C | *ATC1* | Aip Three Complex | 2.79 |
|  | YBR267W | *REI1* | REquired for Isotropic bud growth | 3.18 |
| cellular ion homeostasis | YDR184C | *ATC1* | Aip Three Complex | 2.79 |
|  | YLR083C | *EMP70* |  | 2.38 |
| response to osmotic stress | YLR336C | *SGD1* | Suppressor of Glycerol Defect | 2.74 |
|  | YOR047C | *STD1* | Suppressor of Tbp Deletion | 2.65 |
| response to starvation | YAL036C | *RBG1* | RiBosome interacting Gtpase | 2.72 |
|  | YNL209W | *SSB2* | Stress-Seventy subfamily B | 2.39 |
| protein folding | YBR155W | *CNS1* | CyclophiliN Seven suppressor | 2.55 |
|  | YNL209W | *SSB2* | Stress-Seventy subfamily B | 2.39 |
| mitochondrion organization | YLR355C | *ILV5* | IsoLeucine-plus-Valine requiring | 2.42 |
|  | YMR241W | *YHM2* | Yeast suppressor of HM mutant | 2.78 |
| protein dephosphorylation | YGR123C | *PPT1* | Protein Phosphatase T | 2.89 |
|  | YIR026C | *YVH1* | Yeast vaccinia virus VH1 Homolog | 3.14 |
| protein modification by small protein conjugation or removal | YGR200C | *ELP2* | ELongator Protein | 2.66 |
|  | YDR130C | *FIN1* | Filaments In between Nuclei | 2.53 |
| telomere organization | YML060W | *OGG1* | 8-OxoGuanine Glycosylase/lyase | 2.61 |
|  | YGL169W | *SUA5* | Suppressor of Upstream AUG | 2.36 |
| cellular respiration | YBR238C | *YBR238C* |  | 2.64 |
| vacuole organization | YIR026C | *YVH1* | Yeast vaccinia virus VH1 Homolog | 3.14 |
| monocarboxylic acid metabolic process | YLR134W | *PDC5* | Pyruvate DeCarboxylase | 4.05 |
| invasive growth in response to glucose limitation | YLR083C | *EMP70* |  | 2.38 |
| vitamin metabolic process | YOR095C | *RKI1* | Ribose-5-phosphate Ketol-Isomerase | 3.26 |
| endocytosis | YBR266C | *SLM6* | Synthetic Lethal with Mss4 | 3.52 |
| tRNA aminoacylation for protein translation | YHR020W | *YHR020W* |  | 2.56 |
| rRNA processing | YDR299W | *BFR2* | BreFeldin A Resistance | 3.31 |
| rRNA processing | YPL217C | *BMS1* | BMh Sensitive | 3.31 |
| rRNA processing | YIL096C | *BMT5* | Base Methyltransferase of Twenty five S rRNA 5 | 3.05 |
| rRNA processing | YMR014W | *BUD22* | BUD site selection | 2.49 |
