# Supplementary material for: Physiological and Molecular Characterization of an Oxidative Stress-Resistant Saccharomyces cerevisiae Strain Obtained by Evolutionary Engineering
Source: Front Microbiol. 2022 Feb 24;13:822864. doi: 10.3389/fmicb.2022.822864 (PMC8911705; doi:10.3389/fmicb.2022.822864)
Supplement: Supplementary file 3 [file Table_3.DOCX]

**Table S3** Comparison of the number of up-regulated and down-regulated genes in H7 according to metabolic processes.

| **Metabolic Process Name** | **2-Fold Changed Genes** | **2-Fold Up-regulated Genes** | **2-Fold Down-regulated Genes** |
| --- | --- | --- | --- |
| rRNA processing | 99 | - | 99 |
| ribosomal small subunit biogenesis | 58 | - | 58 |
| response to chemical | 51 | 40 | 11 |
| ribosomal large subunit biogenesis | 50 | - | 50 |
| carbohydrate metabolic process | 48 | 45 | 3 |
| generation of precursor metabolites and energy | 36 | 31 | 5 |
| nucleobase-containing small molecule metabolic process | 36 | 24 | 12 |
| RNA modification | 28 | 2 | 26 |
| transmembrane transport | 26 | 19 | 7 |
| nuclear transport | 25 | 2 | 23 |
| response to oxidative stress | 25 | 23 | 2 |
| cellular amino acid metabolic process | 23 | 12 | 11 |
| ion transport | 23 | 16 | 7 |
| meiotic cell cycle | 22 | 18 | 4 |
| transcription from RNA polymerase II promoter | 22 | 14 | 8 |
| Signaling | 20 | 10 | 10 |
| organelle assembly | 19 | 1 | 18 |
| ribosome assembly | 18 | - | 18 |
| monocarboxylic acid metabolic process | 18 | 17 | 1 |
| cofactor metabolic process | 18 | 15 | 3 |
| organelle fission | 17 | 9 | 8 |
| protein targeting | 16 | 14 | 2 |
| ribosomal subunit export from nucleus | 16 | - | 16 |
| regulation of cell cycle | 15 | 5 | 10 |
| lipid metabolic process | 15 | 11 | 4 |
| cellular respiration | 15 | 14 | 1 |
| tRNA processing | 15 | - | 15 |
| regulation of organelle organization | 14 | 7 | 7 |
| protein phosphorylation | 14 | 10 | 4 |
| mitochondrion organization | 14 | 12 | 2 |
| mitotic cell cycle | 14 | 4 | 10 |
| protein complex biogenesis | 13 | 9 | 4 |
| sporulation | 13 | 10 | 3 |
| conjugation | 12 | 4 | 8 |
| cell wall organization or biogenesis | 12 | 8 | 4 |
| transcription from RNA polymerase I promoter | 12 | - | 12 |
| RNA catabolic process | 11 | 3 | 8 |
| response to osmotic stress | 11 | 9 | 2 |
| oligosaccharide metabolic process | 11 | 11 | - |
| carbohydrate transport | 11 | 11 | - |
| response to heat | 11 | 11 | - |
| protein modification by small protein conjugation or removal | 11 | 9 | 2 |
| cytoskeleton organization | 10 | 5 | 5 |
| regulation of protein modification process | 9 | 4 | 5 |
| protein folding | 9 | 7 | 2 |
| DNA replication | 8 | 2 | 6 |
| DNA recombination | 8 | 6 | 2 |
| peptidyl-amino acid modification | 8 | 1 | 7 |
| protein alkylation | 8 | 1 | 7 |
| chromosome segregation | 8 | 3 | 5 |
| proteolysis involved in cellular protein catabolic process | 8 | 5 | 3 |
| peroxisome organization | 8 | 8 | - |
| cellular response to DNA damage stimulus | 7 | 4 | 3 |
| regulation of DNA metabolic process | 7 | 3 | 4 |
| pseudohyphal growth | 7 | 4 | 3 |
| DNA repair | 6 | 3 | 3 |
| chromatin organization | 6 | 4 | 2 |
| endosomal transport | 6 | 4 | 2 |
| response to starvation | 6 | 4 | 2 |
| nucleobase-containing compound transport | 6 | 1 | 5 |
| invasive growth in response to glucose limitation | 6 | 5 | 1 |
| endocytosis | 6 | 5 | 1 |
| mRNA processing | 5 | - | 5 |
| RNA splicing | 5 | - | 5 |
| snoRNA processing | 5 | - | 5 |
| vacuole organization | 5 | 4 | 1 |
| cytoplasmic translation | 5 | 1 | 4 |
| Golgi vesicle transport | 5 | 5 | - |
| regulation of translation | 5 | - | 5 |
| histone modification | 4 | 2 | 2 |
| membrane invagination | 4 | 4 | - |
| telomere organization | 4 | 2 | 2 |
| membrane fusion | 4 | 4 | - |
| translational initiation | 3 | - | 3 |
| lipid transport | 3 | 3 | - |
| cellular ion homeostasis | 3 | 1 | 2 |
| cytokinesis | 3 | - | 3 |
| transcription from RNA polymerase III promoter | 3 | - | 3 |
| translational elongation | 3 | - | 3 |
| DNA-templated transcription, elongation | 3 | - | 3 |
| protein dephosphorylation | 3 | 1 | 2 |
| amino acid transport | 3 | 3 | - |
| organelle fusion | 3 | 3 | - |
| DNA-templated transcription, termination | 2 | - | 2 |
| cell morphogenesis | 2 | 2 | - |
| cell budding | 2 | - | 2 |
| nucleus organization | 1 | 1 | - |
| organelle inheritance | 1 | 1 | - |
| mitochondrial translation | 1 | 1 | - |
| regulation of transport | 1 | 1 | - |
| protein maturation | 1 | 1 | - |
| vitamin metabolic process | 1 | - | 1 |
| tRNA aminoacylation for protein translation | 1 | - | 1 |
