# Supplementary material for: Physiological and Molecular Characterization of an Oxidative Stress-Resistant Saccharomyces cerevisiae Strain Obtained by Evolutionary Engineering
Source: Front Microbiol. 2022 Feb 24;13:822864. doi: 10.3389/fmicb.2022.822864 (PMC8911705; doi:10.3389/fmicb.2022.822864)
Supplement: Supplementary file 4 [file Table_4.DOCX]

Table S4 Coverage data and quality information for the single nucleotide variation analysis of the evolved strain H7 and the reference strain 905. Total mapped reads, average base coverage depth, 1x, 20 x and 100 x coverage of genome are indicated.

|  | *905* | H7 |
| --- | --- | --- |
| Number of Mapped Reads | 20,972,820 | 20,174,500 |
| Average Base Coverage Depth | 368.1 | 349.1 |
| Uniformity of Base Coverage | 98.36% | 97.96% |
| Genome Base Coverage at 1x | 99.52% | 99.50% |
| Genome Base Coverage at 20x | 99.09% | 99.08% |
| Genome Base Coverage At 100x | 97.37% | 95.54% |
