# Supplementary material for: Physiological and Molecular Characterization of an Oxidative Stress-Resistant Saccharomyces cerevisiae Strain Obtained by Evolutionary Engineering
Source: Front Microbiol. 2022 Feb 24;13:822864. doi: 10.3389/fmicb.2022.822864 (PMC8911705; doi:10.3389/fmicb.2022.822864)
Supplement: Supplementary file 5 [file Table_5.DOCX]

**Table S5** Up-regulated and down-regulated target genes of *NRG1*, their roles and expression level changes.

| **Gene Name** | **Systematic Name of Gene** | **Description** | **Microarray result** | **Fold Change** |
| --- | --- | --- | --- | --- |
| *AMN1* | YBR158W | Protein required for daughter cell separation; multiple mitotic checkpoints, and chromosome stability; expression is induced by the Mitotic Exit Network (MEN) | down | 2.36 |
| *APD1* | YBR151W | Protein of unknown function; required for normal localization of actin patches and for normal tolerance of sodium ions and hydrogen peroxide | up | 1.16 |
| *BOP2* | YLR267W | Protein of unknown function | up | 3.2 |
| *CCP1* | YKR066C | Mitochondrial cytochrome-c peroxidase; degrades reactive oxygen species in mitochondria, involved in the response to oxidative stress | up | 2.48 |
| *CMK2* | YOL016C | Calmodulin-dependent protein kinase; may play a role in stress response | up | 1.93 |
| *CWP1* | YKL096W | Cell wall mannoprotein that localizes to birth scars of daughter cells; linked to a beta-1,3- and beta-1,6-glucan heteropolymer through a phosphodiester bond; required for propionic acid resistance | up | 4.64 |
| *FAF1* | YIL019W | Protein required for pre-rRNA processing; also required for 40S ribosomal subunit assembly | down | 2.62 |
| *GAL7* | YBR018C | Galactose-1-phosphate uridyl transferase; synthesizes glucose-1-phosphate and UDP-galactose from UDP-D-glucose and alpha-D-galactose-1-phosphate in the second step of galactose catabolism | up | 4.22 |
| *GAT4* | YIR013C | Protein containing GATA family zinc finger motifs; involved in spore wall assembly | up | 3.12 |
| *GPT2* | YKR067W | Glycerol-3-phosphate/dihydroxyacetone phosphate sn-1 acyltransferase; located in lipid particles and the ER; involved in the stepwise acylation of glycerol-3-phosphate and dihydroxyacetone in lipid biosynthesis; the most conserved motifs and functionally relevant residues are oriented towards the ER lümen | up | 2.47 |
| *GSY1* | YFR015C | Glycogen synthase; expression induced by glucose limitation, nitrogen starvation, environmental stress, and entry into stationary phase; GSY1 has a paralog, GSY2, that arose from the whole genome duplication; relocalizes from nucleus to cytoplasmic foci upon DNA replication stress | up | 4.76 |
| *HAP4* | YKL109W | Transcription factor; subunit of the heme-activated, glucose-repressed Hap2p/3p/4p/5p CCAAT-binding complex, a transcriptional activator and global regulator of respiratory gene expression; provides the principal activation function of the complex; involved in diauxic shift | up | 1.53 |
| *HER1* | YOR227W | Protein of unknown function; required for proliferation or remodeling of the ER that is caused by overexpression of Hmg2p; may interact with ribosomes, based on co-purification experiments | up | 1.38 |
| *HOR7* | YMR251W-A | Protein of unknown function; overexpression suppresses Ca2+ sensitivity of mutants lacking inositol phosphorylceramide mannosyltransferases Csg1p and Csh1p; transcription is induced under hyperosmotic stress and repressed by alpha factor | up | 2.73 |
| *HSP30* | YCR021C | Negative regulator of the H(+)-ATPase Pma1p; stress-responsive protein; hydrophobic plasma membrane-localized; induced by heat shock, ethanol treatment, weak organic acid, glucose limitation, and entry into stationary phase | up | 2.25 |
| *HXT2* | YMR011W | High-affinity glucose transporter of the major facilitator superfamily; expression is induced by low levels of glucose and repressed by high levels of glucose | up | 4.82 |
| *ICS2* | YBR157C | Protein of unknown function; null mutation does not confer any obvious defects in growth, spore germination, viability, or carbohydrate utilization | up | 1.37 |
| *JID1* | YPR061C | Probable Hsp40p co-chaperone; has a DnaJ-like domain and appears to be involved in ER-associated degradation of misfolded proteins containing a tightly folded cytoplasmic domain; inhibits replication of Brome mosaic virus in S. cerevisiae | up | 3.56 |
| *KTI12* | YKL110C | Protein that plays a role in modification of tRNA wobble nucleosides; protein plays role in tRNA wobble nucleoside modification with Elongator complex; involved in sensitivity to G1 arrest induced by zymocin; interacts with chromatin throughout the genome; also interacts with Cdc19p | down | 2.02 |
| *MNN1* | YER001W | Alpha-1,3-mannosyltransferase; integral membrane glycoprotein of the Golgi complex, required for addition of alpha1,3-mannose linkages to N-linked and O-linked oligosaccharides, one of five S. cerevisiae proteins of the MNN1 family | down | 1.24 |
| *NCE103* | YNL036W | Carbonic anhydrase; metalloenzyme that catalyzes CO_2_ hydration to bicarbonate, which is an important metabolic substrate, and protons; not expressed under conditions of high CO_2_, such as inside a growing colony, but transcription is induced in response to low CO_2_ levels, such as on the colony surface in ambient air; poorly transcribed under aerobic conditions and at an undetectable level under anaerobic conditions; abundance increases in response to DNA replication stress | up | 4.86 |
| *NRG2* | YBR066C | Transcriptional repressor; mediates glucose repression and negatively regulates filamentous growth; activated in stochastic pulses of nuclear localization in response to low glucose | up | 2.52 |
| *PPM1* | YDR435C | Carboxyl methyltransferase; methylates the C terminus of the protein phosphatase 2A catalytic subunit (Pph21p or Pph22p), which is important for complex formation with regulatory subunits; required for methionine to inhibit autophagy and promote growth | up | 2.21 |
| *PPZ2* | YDR436W | Serine/threonine protein phosphatase Z, isoform of Ppz1p; involved in regulation of potassium transport, which affects osmotic stability, cell cycle progression, and halotolerance | up | 1.12 |
| *RCN2* | YOR220W | Protein of unknown function; phosphorylated in response to alpha factor; protein abundance increases in response to DNA replication stress | up | 2.57 |
| *RCR1* | YBR005W | Protein of the ER membrane involved in cell wall chitin deposition; may function in the endosomal-vacuolar trafficking pathway, helping determine whether plasma membrane proteins are degraded or routed to the plasma membrane | up | 1.52 |
| *SED1* | YDR077W | Major stress-induced structural GPI-cell wall glycoprotein; associates with translating ribosomes, possible role in mitochondrial genome maintenance; ORF contains two distinct variable minisatellites | up | 2.15 |
| *SFL1* | YOR140W | Transcriptional repressor and activator; involved in repression of flocculation-related genes, and activation of stress-responsive genes; has direct role in INO1 transcriptional memory; negatively regulated by cAMP-dependent protein kinase A subunit Tpk2p; premature stop codon (C1430T, Q477-stop) in SK1 background is linked to the aggressively invasive phenotype of SK1 relative to BY4741 (S288C) | down | 2.06 |
| *SMT3* | YDR510W | Ubiquitin-like protein of the SUMO family; conjugated to lysine residues of target proteins; associates with transcriptionally active genes; regulates chromatid cohesion, chromosome segregation, APC-mediated proteolysis, DNA replication and septin ring dynamics | up | 1.16 |
| *TDA6* | YPR157W | Putative protein of unknown function; induced by treatment with 8-methoxypsoralen and UVA irradiation; null mutant is sensitive to expression of the top1-T722A allele | down | 1.82 |
| *TIR1* | YER011W | Cell wall mannoprotein; expression is downregulated at acidic pH and induced by cold shock and anaerobiosis; abundance is increased in cells cultured without shaking; member of the Srp1p/Tip1p family of serine-alanine-rich proteins | down | 1.62 |
| *TPO3* | YPR156C | Polyamine transporter of the major facilitator superfamily; member of the 12-spanner drug:H(+) antiporter DHA1 family; specific for spermine; localizes to the plasma membrane | down | 1.65 |
| *USV1* | YPL230W | Putative transcription factor containing a C2H2 zinc finger; mutation affects transcriptional regulation of genes involved in growth on non-fermentable carbon sources, response to salt stress and cell wall biosynthesis | up | 4.53 |
| *UTR2* | YEL040W | Chitin transglycosylase; functions in the transfer of chitin to beta(1-6) and beta(1-3) glucans in the cell wall; similar to and functionally redundant with Crh1; glycosylphosphatidylinositol (GPI)-anchored protein localized to bud neck | down | 1.6 |
| YBL029C-A | YBL029C-A | Protein of unknown function; protein abundance increases in response to DNA replication stress | up | 2.57 |
| YBL029W | YBL029W | Non-essential protein of unknown function | down | 2.06 |
| YGR237C | YGR237C | Putative protein of unknown function | up | 1.07 |
| YIR014W | YIR014W | Putative protein of unknown function; YIR014W is a non-essential gene | up | 2.17 |
| YJL107C | YJL107C | Putative protein of unknown function; expression is induced by activation of the HOG1 mitogen-activated signaling pathway and this induction is Hog1p/Pbs2p dependent; YJL107C and adjacent ORF, YJL108C are merged in related fungi | up | 3.21 |
| YLR012C | YLR012C | Putative protein of unknown function; YLR012C is not an essential gene | up | 5.96 |
| YMR084W | YMR084W | Putative protein of unknown function; YMR084W and adjacent ORF YMR085W are merged in related strains, and together are paralogous to glutamine-fructose-6-phosphate amidotransferase GFA1 | up | 3.07 |
| YMR085W | YMR085W |  | up | 3.13 |
| YOL014W | YOL014W | Putative protein of unknown function | up | 2.18 |
