# Supplementary material for: Physiological and Molecular Characterization of an Oxidative Stress-Resistant Saccharomyces cerevisiae Strain Obtained by Evolutionary Engineering
Source: Front Microbiol. 2022 Feb 24;13:822864. doi: 10.3389/fmicb.2022.822864 (PMC8911705; doi:10.3389/fmicb.2022.822864)
Supplement: Supplementary file 6 [file Table_6.DOCX]

**Table S6** GO-slim process analysis results of the differentially expressed genes that are regulated by *NRG1*.

| **GO-Slim process name** | **Gene Name** |
| --- | --- |
| ***GO-slim process analysis results of the down-regulated genes that are regulated by NRG1*** | |
| biological process unknown | *TDA6, TIR1, YBL029W* |
| protein glycosylation | *MNN1* |
| cell wall organization or biogenesis | *UTR2* |
| rRNA processing | *FAF1* |
| carbohydrate metabolic process | *UTR2* |
| RNA modification | *KTI12* |
| regulation of cell cycle | *AMN1* |
| regulation of organelle organization | *AMN1* |
| organelle fission | *AMN1* |
| cellular ion homeostasis | *SLF1* |
| ribosomal small subunit biogenesis | *FAF1* |
| transcription from RNA polymerase II promoter | *KTI12* |
| regulation of translation | *SLF1* |
| mitotic cell cycle | *AMN1* |
| tRNA processing | *KTI12* |
| ion transport | *TPO3* |
|  |  |
| ***GO-slim process analysis results of the up-regulated genes that are regulated by NRG1*** | |
| biological process unknown | *APD1, BOP2, ICS2, JID1, RCN2, YBL029C-A, YGR237C, YIR014W, YJL107C, YLR012C, YMR084W, YMR085W, YOL014W* |
| response to chemical | *CCP1, HAP4, HSP30, NCE103, NRG2, USV1* |
| response to oxidative stress | *CCP1, HSP30, NCE103* |
| response to osmotic stress | *HSP30, NRG2, USV1* |
| transcription from RNA polymerase II promoter | *HAP4, NRG2, USV1* |
| cell wall organization or biogenesis | *CWP1, SED1* |
| carbohydrate metabolic process | *GAL7, GSY1* |
| generation of precursor metabolites and energy | *GSY1, HAP4* |
| carbohydrate transport | *HXT2* |
| pseudohyphal growth | *NRG2* |
| protein phosphorylation | *CMK2* |
| lipid metabolic process | *GPT2* |
| cellular respiration | *HAP4* |
| protein alkylation | *PPM1* |
| cellular response to DNA damage stimulus | *HSP30* |
| protein complex biogenesis | *PPM1* |
| invasive growth in response to glucose limitation | *NRG2* |
| sporulation | *CWP1* |
| peptidyl-amino acid modification | *SMT3* |
| mitochondrion organization | *SED1* |
| response to heat | *HSP30* |
| signaling | *CMK2* |
| meiotic cell cycle | *CWP1* |
| cellular ion homeostasis | *PPZ2* |
| protein modification by small protein conjugation or removal | *SMT3* |
|  |  |
| ***cannot be mapped to a GO slim term*** | |
| *GAT4, HER1, HOR7, RCR1* | |
